# Plant Growth Promotion and Heat Stress Amelioration in Arabidopsis Inoculated with *Paraburkholderia phytofirmans* PsJN Rhizobacteria Quantified with the GrowScreen-Agar II Phenotyping Platform

**DOI:** 10.3390/plants11212927

**Published:** 2022-10-30

**Authors:** Allene Macabuhay, Borjana Arsova, Michelle Watt, Kerstin A. Nagel, Henning Lenz, Alexander Putz, Sascha Adels, Mark Müller-Linow, Jana Kelm, Alexander A. T. Johnson, Robert Walker, Gabriel Schaaf, Ute Roessner

**Affiliations:** 1 School of BioSciences, University of Melbourne, Parkville, VIC 3010, Australia; 2 Institute for Bio- & Geosciences (IBG-2), Plant Sciences, Forschungszentrum Juelich GmbH, 52425 Juelich, Germany; 3 Institute of Crop Science and Resource Conservation, Department of Plant Nutrition, University of Bonn, 53115 Bonn, Germany; 4 Research School of Biology, The Australian National University, Acton, ACT 2601, Australia

**Keywords:** plant growth-promoting rhizobacteria (PGPR), *Paraburkholderia phytofirmans* PsJN, phenotyping, root morphology, root system architecture, high temperature, *Arabidopsis thaliana*, growth stimulation, heat tolerance

## Abstract

High temperatures inhibit plant growth. A proposed strategy for improving plant productivity under elevated temperatures is the use of plant growth-promoting rhizobacteria (PGPR). While the effects of PGPR on plant shoots have been extensively explored, roots—particularly their spatial and temporal dynamics—have been hard to study, due to their below-ground nature. Here, we characterized the time- and tissue-specific morphological changes in bacterized plants using a novel non-invasive high-resolution plant phenotyping and imaging platform—GrowScreen-Agar II. The platform uses custom-made agar plates, which allow air exchange to occur with the agar medium and enable the shoot to grow outside the compartment. The platform provides light protection to the roots, the exposure of it to the shoots, and the non-invasive phenotyping of both organs. *Arabidopsis thaliana*, co-cultivated with *Paraburkholderia phytofirmans* PsJN at elevated and ambient temperatures, showed increased lengths, growth rates, and numbers of roots. However, the magnitude and direction of the growth promotion varied depending on root type, timing, and temperature. The root length and distribution per depth and according to time was also influenced by bacterization and the temperature. The shoot biomass increased at the later stages under ambient temperature in the bacterized plants. The study offers insights into the timing of the tissue-specific, PsJN-induced morphological changes and should facilitate future molecular and biochemical studies on plant–microbe–environment interactions.

## 1. Introduction

Plants are constantly challenged by an array of biotic and abiotic constraints that limit their growth and productivity. These include environmental stresses such as drought, flooding, extreme temperatures, metal toxicity, and nutrient deficiency, as well as exposure to phytopathogens and herbivorous insects [1].

Climate-related changes, in particular rising global temperatures, are a serious environmental challenge and they are of major concern for future crop production. Temperature plays essential roles in all of the stages of plant development and any exposure outside the optimal range can be stressful or lethal [2]. Plant responses to cope with heat stress vary with the intensity and duration of the elevated temperatures [3]. Aside from directly affecting the plants, increased global temperatures also aggravate other existing abiotic stressors such as salinity, drought, or mineral toxicity [4]. Two general mechanisms for surviving high temperature conditions are employed by plants. Thermomorphogenesis (responses that occur at moderately high temperatures [5]) induces short-term avoidance, e.g., the elongation of the primary roots in search of cooler soil area and water [6,7,8] or acclimation, e.g., the changing of the leaf orientation and transpirational cooling through the elongation of the hypocotyl, petioles, and leaves [9,10,11]. On the other hand, plants can respond with long-term evolutionary phenological and morphological adaptations [12,13] that are associated with heat stress. Heat stress occurs when the rise in temperature for a certain duration exceeds a species-specific threshold level [2] that is sufficient to cause irreversible damage to the plants’ growth [14].

Plants’ morphological responses to elevated temperatures are very dynamic [15]. Developmentally, these growth parameters are revealed over the lifetime of a plant through various cellular processes, e.g., cell division, cell expansion, and anisotropic growth [15,16]. While high temperatures have diverse morphological effects on the aboveground plant tissues, such as the scorching of the twigs and leaves along with visual symptoms of sunburn, the senescence of leaves, growth inhibition, and the discoloration of fruits [17]; belowground, they also cause significant modifications to the root system [18]. A moderately elevated temperature leads to root elongation, but this effect stops around 30 °C, after which the root length decreases [19]. This is reflected in a decreased amount of root growth rate, a decreased meristematic zone at 30 °C when it is compared to those at 20 °C or 25 °C, and a decreased number of cells in the elongation zone of the root. Roots that are grown at 30 °C also have smaller radii than roots that are grown at 20–25 °C [19]. Furthermore, another strategy in the RSA to cope with increasing temperatures is the increase in the number and length of the root hairs, which enhances the root surface area for an improved exploration of the soil for water and nutrients [20].

Elevated temperatures often have cumulative effects which result in poor plant growth and performance [4]. Consequently, we require novel approaches to mitigate the plant stress response and meet the agricultural goals under elevated temperatures [21]. Several strategies are already in practice to develop heat-tolerant crop plants, including conventional crop breeding and genetic engineering approaches [22,23].

The use of plant growth-promoting rhizobacteria (PGPR), in addition to the above, is another strategy that can contribute to agricultural sustainability [24,25].

Unlike obligate symbionts, PGPR can interact with numerous hosts and improve plant health and stress tolerance through a multitude of mechanisms [26,27,28]. PGPR are capable of modulating the root system architecture (RSA), which can significantly affect the crop performance and productivity [29,30,31]. The RSA refers to the integrated root system topology, the spatial distribution of the primary and lateral roots, and the number and length of various root types. The RSA is modified by PGPR mainly through their ability to interfere with the plant’s hormonal balance and processes [32]. The implication of the species-specific production of phytohormones, secondary metabolites, and enzymes leads to changes in the growth rates of the primary roots, the branching of the lateral roots, and the density of the root hairs [32,33].

In the present context of rising global temperatures, it is useful to study PGPR which also have thermotolerant traits. In the past, several thermotolerant microbial strains were identified [24,34], including Burkholderia phytofirmans [35]. Paraburkholderia, which is a more recently described new genus that is delineated from Burkholderia, contains many species that assist the plants’ growth [36,37]. *Paraburkholderia phytofirmans* PsJN is a well-studied model PGPR that is known to induce tolerance to both biotic and abiotic stresses. This beneficial bacterial endophyte is capable of colonizing a wide range of plants including wheat [38], maize [39], grapevines [40], tomatoes [41], and potatoes [35]. Its plant-beneficial effects are attributed to several mechanisms including the production or modulation of plant phytohormones [42], the facilitation of resource acquisition [38], the production of siderophores and secondary metabolites [37], and the induction of systemic resistance (ISR) [43]. PsJN has been used in different plant studies against drought, low temperatures, and salinity [38,44,45]. Generally in the root, PsJN has increased the root biomass in maize, potatoes, Brassica and switchgrass, and in the latter two, it also promoted root elongation. It has been shown to increase the number of root hairs in several species including Arabidopsis [46]. To our knowledge, however, only two studies to date have utilized this bacterium to protect against high-temperature stress, and these have focused on the genotypic responses of potatoes [41] as well as the physiological and biochemical changes in tomato aerial tissues [47]. In potatoes [41], PsJN generally increased the root dry weight at the elevated temperatures of 18 potato clones when they were compared to the non-bacterized plants. To our knowledge, the question of the adaptation of the root system architecture to an elevated temperature when the plants are bacterized with PsJN is still open.

Quantifying plant growth dynamics requires non-invasive phenotyping on the same plant individuals through time. While multiple approaches exist for the aerial part of the plant, the roots represent a particular challenge due to their hidden nature (inherently, they are in the soil). Earlier investigations of plants’ roots and their interactions with microorganisms only utilized destructive measurements or harvest sampling methods (e.g., [48]). Destructive approaches discriminate and fail to capture the diverse growth patterns and dynamics within a plant’s ontogeny. To enable an ontogenetic and non-destructive approach, several phenotyping platforms have been employed in plant studies to characterize the root traits [49]. Now, non-invasive, time-resolved phenotyping is becoming more common to the study of plant root-microorganism processes [31,50].

The root phenotyping pipelines consist of a plant growth system, root imaging, and root trait digitization [51]. A traditional plant cultivation practice to quantify the root growth parameters is the use of a transparent medium such as agar gels [52]. Since manual measurements of the root traits of agar-grown plants are labor- and time-intensive, there is a necessity for increasing the throughput. This is addressed with the use of camera- or scanner-based imaging with varying degrees of automation [52], such as the use of multiple scanners operating in parallel [53,54] or the use of a shifting camera that is operated by moving stages or a robotic gantry system [55,56,57]. Here, we used the 2D imaging system of the novel GrowScreen-Agar II platform and its associated image analysis software tools, which were specifically designed for the high-throughput and non-destructive phenotyping of root and shoot development through time. This platform also includes an optimized agar-plate cultivation with the shoots growing outside the plate and with the roots kept in the dark to avoid the occurrence of light-related responses, while providing air exchange to the agar surface [58]. Moreover, the GrowScreen-Agar II allows for the observation of the plants’ responses which are subjected to one or more environmental factors.

This study quantifies the growth stimulation that is imparted by the plant growth-promoting rhizobacteria *P. phytofirmans* PsJN through the investigation of the morphologic and dynamic responses of Arabidopsis roots and shoots which were measured in a three-week period. Additionally, this study examines how the application of the beneficial rhizobacteria can ameliorate the detrimental effects of a constantly high temperature. In doing so, we provide novel insights on time-specific phytomorphological responses to bacterial inoculation, which enhance the growing knowledge on PGPR for their application in agriculture under future high-temperature climate conditions. Finally, we describe the technology that can drive knowledge generation by encompassing plant developmental dynamics in plant–microbe–environment interactions.

## 2. Results

### 2.1. Increased Plant Growth and Higher Plant Biomass at 21 Days Post-Inoculation with Bacteria PsJN in a “Closed-Plate” System

Our pilot experiment utilizing the traditional closed-plate system showed that PsJN promote the growth and heat tolerance to Arabidopsis plants. For the control plants, a high temperature resulted in a decrease in the total root length, with this reduction ranging from 21% (at 2 DAI) to 76% (at 21 DAI) (Figure S1a). When the total root length was discriminated into the primary and branched root components, we found a similar decline in the root lengths due to the high temperature. The primary root lengths showed a reduction that was between 21% and 48%, while the branched roots showed a larger amount of reduction that was between 52% and 77% from 2 to 21 DAI, indicating the strong effect of a high temperature on the latter root types (Figure S1b,c).

Although the increased temperature detrimentally affected the root lengths, this was ameliorated by the inoculation of them with PsJN, the magnitude of which varied in the root types. At 21 DAI and at a high temperature, the PsJN-inoculated plants showed a 123% increase in total root lengths when they were compared to the control plants (Figure S1a). Interestingly, although this positive bacterial inoculation effect on the primary root was strong at the start of the experiment (at 52%), this declined towards the end of the growing period. The branched roots also showed the same decreasing inoculation effect, which was from 225% to 136% (Figure S1b,c). Under the ambient temperature at 21 DAI, the bacterial inoculation increased the total root lengths by 37% from that of the control plants, and this was mainly observed in the branched roots. However, this positive effect of bacterial inoculation on the total root lengths under the ambient temperature, was 3.3 times lower than when it was compared to its effect under the high temperature (Figure S1a).

The root and shoot weights were reduced by the high temperature and the ambient temperature (at 50% and 20% reduction rates, respectively) (Figure S1d,e). The bacterial inoculation minimized this reduction under the high temperature by increasing the root weights by 6% and the shoot weights by 5% when they were compared to the control plants. However, a stronger effect of bacterial inoculation was observed under the ambient temperature, with an increase of 42% and 16% for the root and shoot weights, respectively (Figure S1d,e).

The conventional “closed-plate” system, which was coupled with the WinRhizo imaging platform, allowed the plant-bacteria co-cultivation and periodic imaging and trait analysis to be performed (Figure S1). However, this system did not allow the natural growth of the shoots and roots, but instead, it enclosed the whole plant inside the plate, providing restrictions on the plant’s development. In addition, the software analysis tool was not sensitive enough to accurately discriminate between the higher order root types (1st- and 2nd-order lateral), particularly at the later stages of the roots’ growth. Moreover, it did not enable us to save the previously analyzed root structures for them to be appended for the succeeding growth stage analysis, which provides a more efficient and accurate way of monitoring the root morphologic growth.

### 2.2. In-Depth Root Characterization Shows PsJN-Imparted Growth Promotion on Root System Architecture (RSA) as Influenced by Temperature and Time

**Figure 1:**
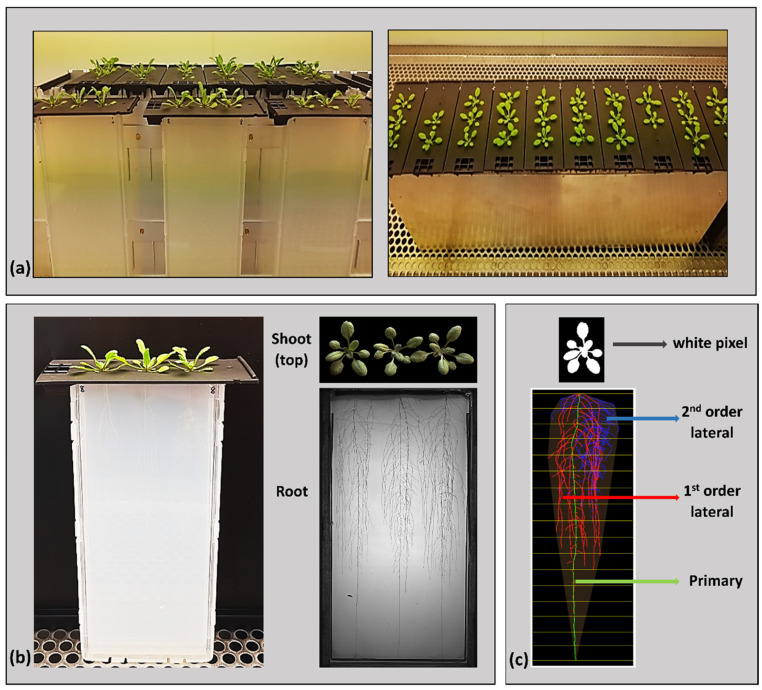
**GrowScreen-Agar II: Plant cultivation setup and root/shoot image acquisition and analysis.** (**a**) Arabidopsis plants growing in customized plates and magazine (product specification on Figures S1 and S2, respectively). Black plate collars provide the background for the growing shoots outside of the plates, while at the same time they exclude light entering the top of the metal magazine (**bottom** panel) to provide dark environment for the roots. (**b**) Overview of a single plate that is manually transferred to an imaging system (Figure S3) equipped with cameras for imaging the shoots and the roots, generating two images: top view of the shoot(s) and the whole root system. (**c**) (**Top**) Analysed image of a rosette using the Colour segmentation tool that computes the sum of white pixels as a proxy to compute the projected leaf area [59]. (**Bottom**) Analysed image of a root system using the GrowScreen-Root software [52]. Different root types are distinguished by color: green for primary roots, red for 1st-order lateral roots, and blue for 2nd-order lateral roots. The whole image area can also be divided horizontally into several sections to extract root length density per layer.

The GrowScreen-Agar II platform addressed all of the issues of the traditional “closed plate” approach. First, the customized plates for the imaging system allowed the unobstructed growth of the rosette and the inflorescence (Figure 1a) due to there being open holes. The plates were custom-made for the imaging system for the plants to be consistently positioning which is critical for the consequential imaging and image superimposition during the analysis stage. The infrared light that was used for the root imaging prevented the disturbance or damage occurring to the root tissues. The depth of the system was increased by almost 100% from the closed plates to a maximal 20 cm depth. As a result, the Arabidopsis plants grew well, simulating the unobstructed upward growth of the shoots that were exposed to light and air, and the undisturbed downward growth of the roots that were in the dark. The dark environment ensured that no skewing occurred during the gravitropic growth process (Figure 1b). Second, with the associated image analysis software, the Colour segmentation tool (shoot) and GrowScreen-Root (root), several traits were quantitively measured; in particular for the roots, which is normally a challenge in a time-course study (Figure 1c). The GrowScreen-Agar II platform also provided an opportunity to further investigate the architecture of the root system by separating the branched roots into 1st- and 2nd-order lateral roots and by measuring other quantifiable spatial and temporal parameters, thereby highlighting the effects of bacteria under two different temperatures. The platform is suitable for plant–microbe interaction studies, as we could confirm that PsJN which was inoculated at the germinated seed stage was also found in the root-tip regions of the 21-day-old plants (Figure S2), while at the same time, the plates looked clean and did not show any signs of colony growth on the agar (Figure S3).

**Figure 2:**
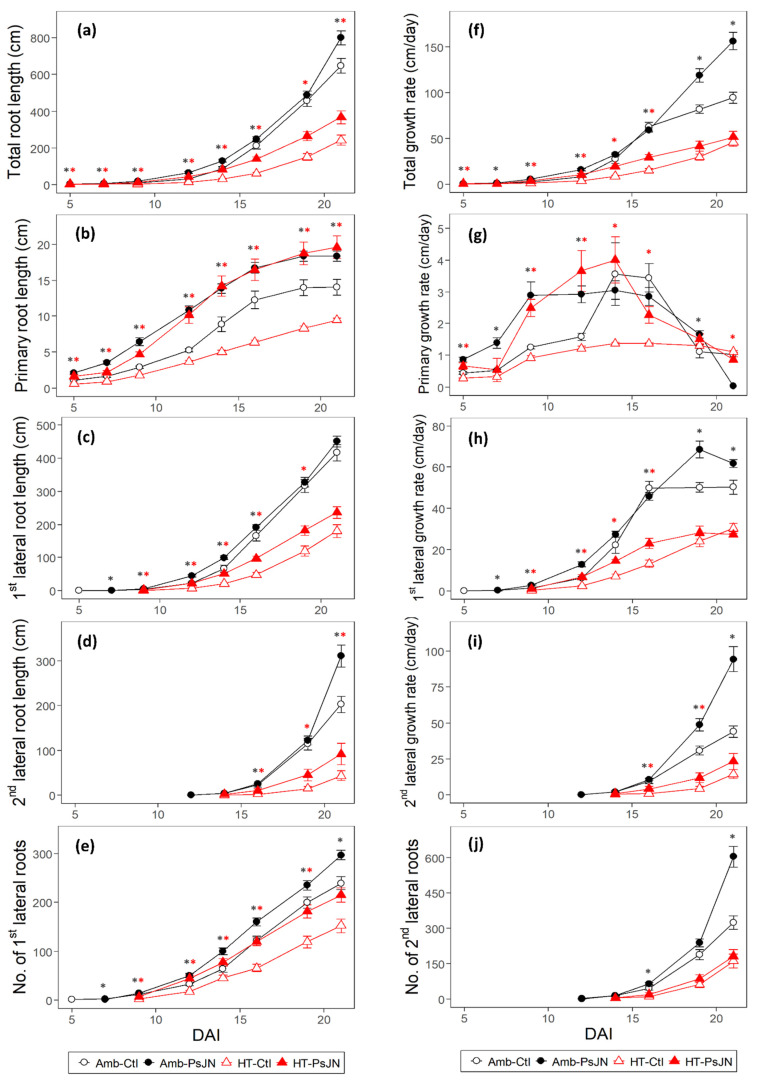
**Morphological traits of different root types.** Traits were measured in plants with or without bacteria PsJN inoculation under ambient or high temperature conditions from 5 to 21 days after inoculation (DAI) in the GrowScreen-Agar II platform. Root lengths: (**a**) total, (**b**) primary, (**c**) 1st-order lateral roots, (**d**) 2nd-order lateral roots; Growth rates: (**f**) total, (**g**) primary, (**h**) 1st-order lateral roots, (**i**) 2nd-order lateral roots; Number of lateral roots: (**e**) 1st-order lateral roots, (**j**) 2nd-order lateral roots. Temperature—black and circle symbol (ambient), red and triangle symbol (high temperature); Bacterial application—empty symbol (control), filled symbol (PsJN-inoculated). Treatments: Amb-Ctl (control plants under ambient temperature), Amb-PsJN (PsJN-inoculated plants under ambient temperature), HT-Ctl (control plants under high temperature), and HT-PsJN (PsJN-inoculated plants under high temperature). All of the points are the mean ± standard error of *n* = 12 (Amb-Ctl), 15 (Amb-PsJN), 12 (HT-Ctl), 8 (HT-PsJN) samples within each treatment. Asterisks: Black—significant difference between mean of PsJN-inoculated and control plants under ambient condition, red—significant difference between mean of PsJN-inoculated and control plants under high temperature condition; these were based on the Student’s *t*-test with *p* < 0.05.

The high temperature decreased the total root lengths by 63% from that of the ambient temperature condition, although with them being inoculated, the decrease of this was smaller at 43% (Figure 2a). The PsJN application increased the total root lengths of the plants under both the ambient and high-temperature conditions, and this was shown to occur consistently from 5 DAI up to 21 DAI (Figure 2a). The difference between the inoculated and control plants peaked at 12 DAI by 103% for the ambient temperature and 202% for the high temperature, before slowly dropping after this. At the end of the growing period, under the high temperature, the bacterial inoculation increased the total root lengths by 52% from that of the control plants. This was two times more than the increase that was observed under the ambient temperature (Figure 2a, Table S1). The total root growth rates also indicated the same trend, with the high temperature causing a 51% reduction from that of the ambient temperature condition (Figure 2f). Under the high temperature, the inoculated plants performed better than the control plants did, with there being an increase of between 178% and 227% from 5 to 9 DAI. On the other hand, this effect was lower, and it was between 92% and 121% under the ambient temperature. Subsequently, the difference in the growth rates of the inoculated and control plants declined such that at 21 DAI, the inoculated plants displayed only a 65% and a 12% increase under the ambient and high temperatures, respectively (Figure 2f, Table S1).

To better understand the RSA, we analyzed the roots according to their root types. The primary roots were negatively affected by the temperature, with the high temperature causing an average length reduction of 41% from that of the ambient temperature throughout the growth period (Figure 2b). The primary roots also responded to the bacterial inoculation at both of the temperature regimes with varying magnitudes. Under the high temperature, the primary root lengths of the inoculated plants maintained an average increase of 154% from that of the control plants; while at the ambient temperature, the stimulation effect of the bacteria decreased from 121% after 9 DAI Figure 2b). At the end of the growing period, the difference in the primary root lengths between the inoculated and the control plants was 3.5 times higher under the high temperature (107%) than it was in the ambient condition (31%). Furthermore, under the ambient condition, the development of the primary roots of both the inoculated and control plants, plateaued after 19 DAI (maximal length of 20 cm as with the plate size), whereas, both the inoculated and control plants showed a linear increase until the end of the experiment under the high-temperature conditions (Figure 2b, Table S1). The growth rates of primary roots also showed a response to the temperature, with the high temperature causing an average reduction of 43% from that of the ambient temperature (until 16 DAI) (Figure 2g). The bacterial inoculation under the high temperature countered this through the stimulation of an increase in the primary root growth rates up until 14 DAI, before the root growth rates rapidly declined. Under the ambient condition, the bacterial application also increased the primary root growth rates; however, the growth rates of the inoculated plants did not change from 9 DAI until 14 DAI, when they started declining (Figure 2g, Table S1).

The deleterious effect of the high temperature was especially evident in the length of the 1st-order laterals (from 9 to 21 DAI), with the heat-stressed plants showing an average reduction of 70% from that of the ambient plants (Figure 2c). The inoculated plants showed an increase in the length of the 1st-order laterals under the high-temperature condition. This inoculation effect was present throughout the growth period, although the stimulation effect declined right from the onset of their growth (from 584% to 31%). Under the ambient condition in the beginning, there was increased growth until 12 DAI (103%), after which the stimulation effects decreased (Figure 2c, Table S1). The high temperature caused a decrease in the growth rates of the 1st-order lateral roots from that of the ambient temperature condition, with an average of 65% (Figure 2h). The increase in the growth rates of the inoculated plants at the high temperature was 584% at 9 DAI, but that declined down to 17% at 19 DAI (Table S1). The control plants, however, showed continuous linear growth rates. At the ambient temperature, the PsJN inoculation caused an increase in the growth rates from that of the control plants up until 12 DAI (by 103%), then this declined afterward. Whilst the control plants showed increasing growth rates until 16 DAI, when their growth rates became constant, the inoculated plants showed an almost linear growth up until 19 DAI, before their growth declined (Figure 2h). The number of 1st-order lateral roots was reduced in the high temperature condition by an average of 47% from that of the ambient temperature condition (Figure 2e). The bacterial inoculation also influenced the number of the 1st-order laterals, with there being increased numbers from those of the control plants which started to appear at 9 DAI: a 275% increase under the high temperature and a 32% increase under the ambient temperature. At the end of the growing period, the difference in the number of 1st-order lateral roots between the inoculated and control plants under the high temperature was twice that of the ambient temperature. This illustrates the strong involvement of the bacteria PsJN in the emergence of these root parts at both of the temperatures (Figure 2e, Table S1).

Upon the emergence of the 2nd-order lateral roots, the high temperature caused a reduction of 95% (6.5 cm) in the length of these root types, which decreased to 79% (308 cm) at 21DAI (Figure 2d). The positive effect of the inoculation under the high temperature (from 16 DAI) led to about 6.5 times longer 2nd-order lateral root lengths than those of the control plants (at 9.7 vs. 1.5 cm, respectively). At the same timepoint, this bacterial stimulation effect was weaker under the ambient temperature, with the inoculated plants (25 cm) being only about 1.1 times taller than the control plants (22.5 cm). At the end of the growing period, the absolute difference in the 2nd-order lateral root lengths between the heat-stressed inoculated and control plants was 48.5 cm, while under the ambient condition, it was 108.5 cm (Figure 2d, Table S1). The high temperature significantly reduced the 2nd-order lateral root growth rates, although this negative effect weakened over time (Figure 2i). At 14 DAI, the high temperature caused a reduction in growth rate of about 18.5 times, whilst at 21 DAI, this reduction was only three times from that of the ambient condition. Throughout the growing period, the growth rate stimulation by the bacteria decreased under the high temperature (from 8.3 down to 1.6 times), while it was doubled under the ambient condition. At 21 DAI, the ambient-grown plants had the highest growth rates of the 2nd-order laterals, with those of the inoculated and control plants being 94 and 44 cm/day, while those of the heat-stressed plants were 23 and 14 cm/day, respectively (Figure 2i, Table S1). Similarly, the number of 2nd-order lateral roots was also decreased by the high temperature by seven times (14 DAI) to two times (21 DAI) lower that of the ambient temperature (Figure 2j). Surprisingly, the effect of the bacterial inoculation was only significant under the ambient temperature (16 and 21 DAI). In the end, the positive effect of the bacterial inoculation on this root type was quite apparent under the ambient condition with an 86% increase in them when they was compared to the control ones. This difference between the inoculated and control ones is about seven times more than it was in the high-temperature condition at 12% (Figure 2j, Table S1).

### 2.3. High Temperature Negatively Impacts the Root System Distribution, but PsJN Buffers This Effect through Improvements to Individual Root Types

**Figure 3:**
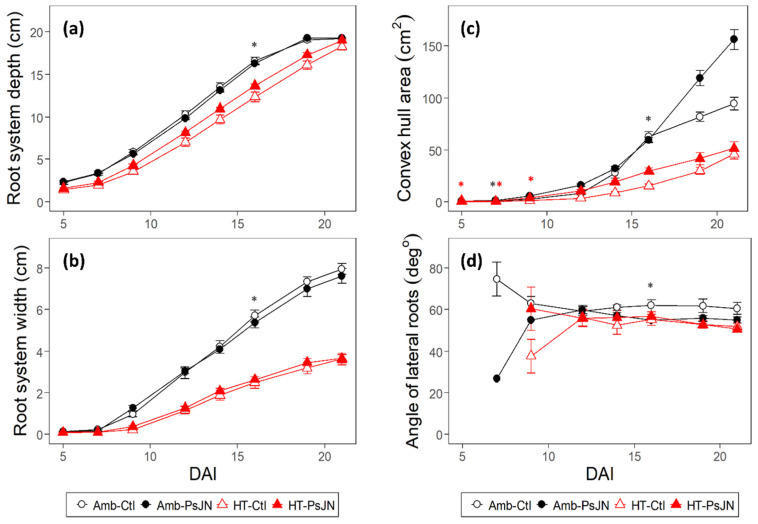
**Root system morphological traits.** Traits were measured from plants with or without bacteria PsJN inoculation under ambient or high temperature conditions from 5 to 21 days after inoculation (DAI) in the GrowScreen-Agar II platform. (**a**) Root system depth, (**b**) root system width, (**c**) convex hull area, and (**d**) branching angle of 1st-order lateral roots. Temperature—black and circle symbol (ambient temperature), red and triangle symbol (high temperature); Bacterial application—empty symbol (control), filled symbol (PsJN-inoculated). Treatments: Amb-Ctl (control plants under ambient condition), Amb-PsJN (PsJN-inoculated plants under ambient condition), HT-Ctl (control plants under high temperature), and HT-PsJN (PsJN-inoculated plants under high temperature). All of the points are the mean ± standard error of *n*= 12 (Amb-Ctl), 15 (Amb-PsJN), 12 (HT-Ctl), 8 (HT-PsJN) samples within each treatment. Asterisks “*”: Black—significant difference between mean of PsJN-inoculated and control plants under ambient condition, red—significant difference between mean of PsJN-inoculated and control plants under high temperature condition; these were based on the Student’s *t*-test with *p* < 0.05.

The two temperature regimes showed clear differences in the root system distribution, with the ambient-grown plants showing greater depths to them than those of the heat-stressed plants did (Figure 3a). In general, the high temperature prevented the downward elongation of the roots, causing an average reduction of 28% from those of the ambient temperature condition. The difference between the two temperature regimes, i.e., Amb-Ctl and HT-Ctl, decreased after 16 DAI, which can be attributed to the faster growth rates of the primary and 1st-order lateral roots under the ambient temperature (Figure 2g,h) that became constant towards the last two timepoints, presumably due to the limitation of the plate. In addition, the slower but continuous growth, particularly the linear growth, of the 1st-order lateral roots under the high temperature (Figure 2g,h) towards the end of the growing period also reduced the difference in the depths of the heat-stressed and ambient-grown roots (Figure 3a). The high temperature also reduced the convex hull area and the branching angle of the root system by an average of 69% and 27%, respectively (Figure 3c,d). The reduction in the root system width due to the high temperature may be attributed to the responses of the 1st- and 2nd-order lateral roots (Figure 2c,d,h,i), which influence the horizontal extension of the root system. Moreover, the decrease in the number (Figure 2e,j), length (Figure 2c,d), growth rate (Figure 2h,i), and the angle of the growth (Figure 3d) of these lateral roots may have contributed to the huge difference between the root system width of the heat-stressed and ambient-grown plants (Figure 3b).

In general, the effect of bacterial inoculation only manifested at 16 DAI under ambient temperature on the overall root system depth (Figure 3a), width (Figure 3b), and branching angle (Figure 3a,b,d). Whilst bacterial effects in these cumulative root traits were not found, it could be because there was compensation between the various root types and root traits (Figure 2). The convex hull area showed a significant bacterial effect under ambient at 7, whereas this effect was evident under high temperature from 5 to 9 DAI (Figure 3c).

### 2.4. Root Length Per Depth Is Influenced over Time by Bacterial Inoculation and High Temperature

**Figure 4:**
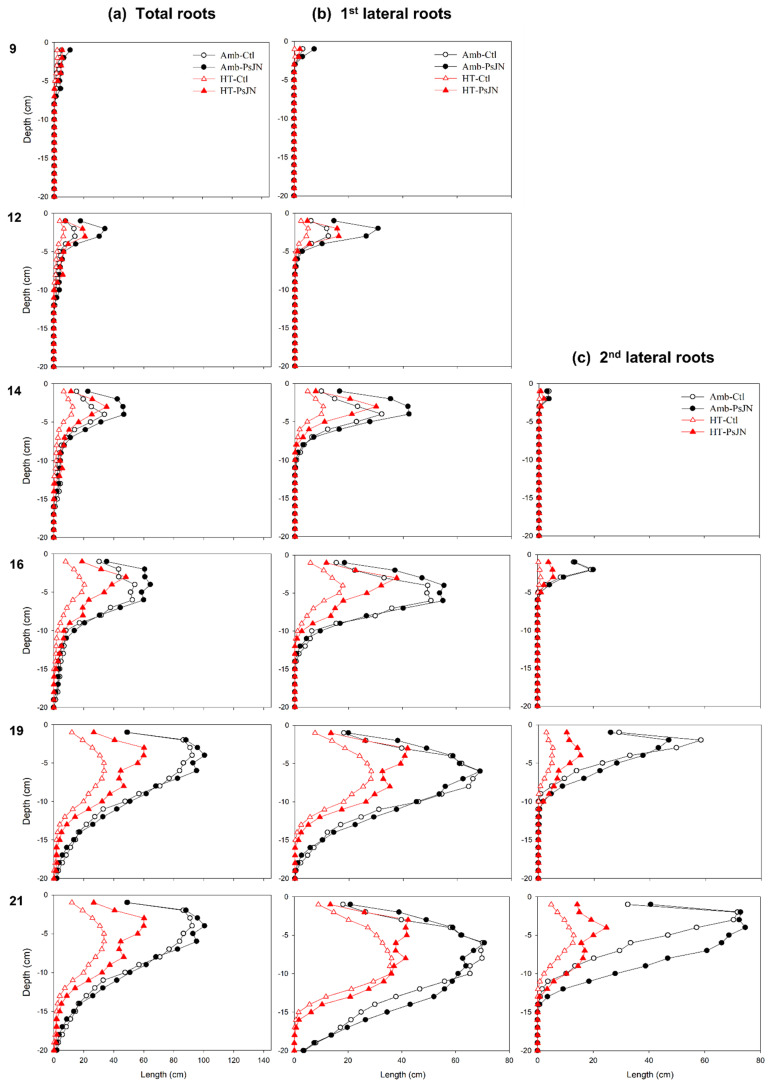
**Root length distribution across different depths through time.** Length distribution of plant roots from all of the treatments across different depths or layers (20 horizontal sections) at several timepoints (9, 12, 14, 16, 19, and 21 DAI) for total root length (**a**); First order lateral roots (**b**) Second order lateral roots (**c**). Treatments: (black points and open symbols)—Amb-Ctl (control plants under ambient condition), (black points, closed symbols)—Amb-PsJN (PsJN-inoculated plants under ambient condition), (red points, open symbols)—HT-Ctl (control plants under high temperature), and (red points, closed symbols)—HT-PsJN (PsJN-inoculated plants under high temperature). All of the points are the mean ± standard error of *n*= 12 (Amb-Ctl), 15 (Amb-PsJN), 12 (HT-Ctl), 8 (HT-PsJN) samples within each treatment.

The depth of the agar plate was divided into 20 horizontal layers, which are referred to here as “depths” from the top or exposed agar surface (0 cm), which divides the root and shoot. The software allowed us to extract the exact length of the primary and lateral roots in each of these “depths” and to evaluate the system distribution across the root types over depth and time (Figure 4). The plants from all of the treatments started growing lateral roots from 5 DAI, but a large difference between the treatments was not found until 9 DAI when the inoculated plants under the ambient condition showed the highest total root length (5.6 cm) which was initially 1 cm below the open-top plate surface (Figure 4a). At 12 DAI, the effect of bacterial inoculation already started manifesting under the high temperature as well at the depths between 1 and 4 cm. Shortly after (from around 14 to 16 DAI), the high temperature started decreasing the root length distribution from 4 to 8 cm. At the same timepoints, the bacterial stimulation effect on the total root lengths from the upper depths (1 to 9 cm) increased under both of the temperature regimes (Figure 4a). This effect was also shown in the 1st-order lateral root lengths (Figure 4b). For the 2nd-order laterals, the effect of the inoculation was first observed at 16 DAI under the high temperature between 1 and 3 cm (Figure 4c). At 19 DAI, the stimulation effect of the bacteria on the root lengths (at 1 to 10 cm depth) were more pronounced under the high temperature when they were compared to those under the ambient temperature (Figure 4a). This can be attributed to the positive responses of both 1st- and 2nd-order laterals under the high temperature (Figure 4b,c). At the end of the growing period (21 DAI), the effect of the inoculation on the total root lengths under both the ambient and high-temperature condition was prominent from the top of them down to a 15 cm depth (Figure 4a). This effect was mainly due to the differential effects of the two lateral roots (Figure 4b,c). While the effect of the bacteria on the 1st-order laterals diminished under the ambient condition, its effect on the 2nd-order laterals increased towards the lower depths (from 5 to 13 cm). On the other hand, under the high temperature, the bacterial inoculation caused the 1st- and 2nd-order lateral roots to display greater root length distributions than the control plants did, with an almost bimodal curve (Figure 4b,c).

Overall, these results show that at all of the stages and root types, the bacterial inoculation increased the root length per depth under both of the temperatures. While the effect of the inoculation for the 1st-order lateral roots under the ambient condition declined through time, the opposite was found for the late-emerging 2nd-order laterals. This mainly accounted for the difference between the inoculated and control under the ambient condition. On the other hand, the effect of the bacteria under the high temperature can be summed up from the combined effects of the 1st- and 2nd-order laterals.

### 2.5. Growth Associations between Root Types Vary under Each Treatment

**Figure 5:**
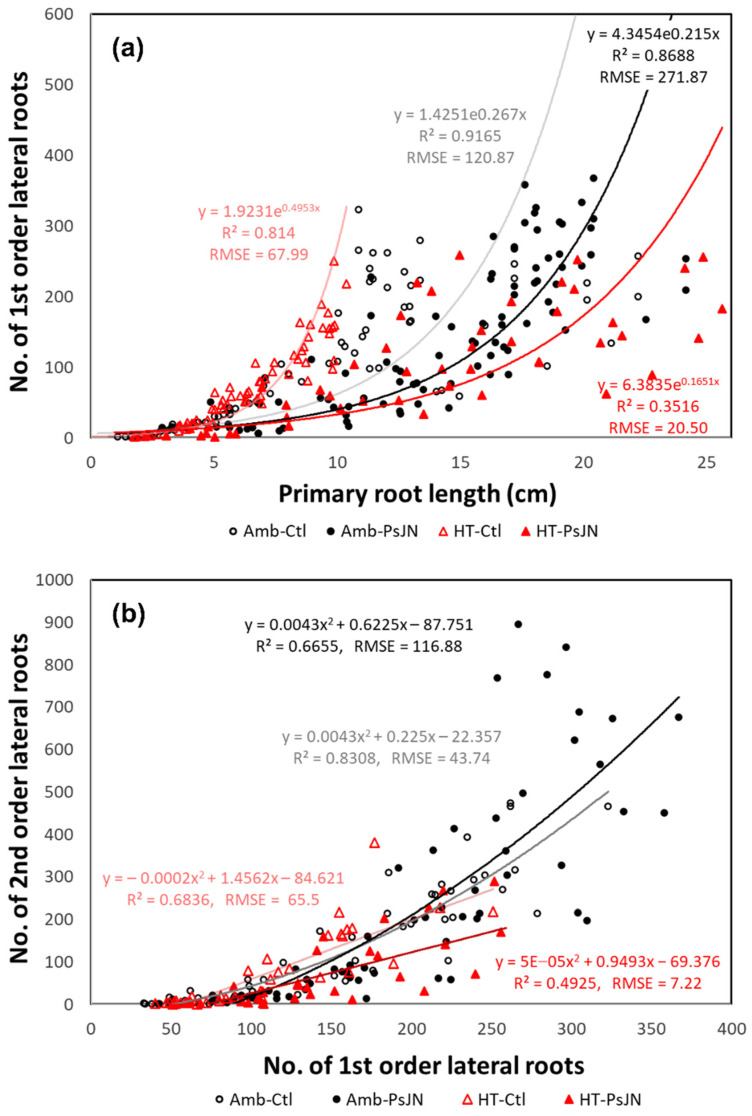
**Correlation analysis for lateral roots**. (**a**) Correlation between the length of the primary roots (Figure 2b) and the number of 1st-order lateral roots (Figure 2e). (**b**) Correlation between the number of 1st- and 2nd-order lateral roots of plants from all of the treatments. Treatments: Amb-Ctl (control plants under ambient condition)—grey, empty circle; Amb-PsJN (PsJN-inoculated plants under ambient condition)—black, filled circle; HT-Ctl (control plants under high temperature)—pink, empty triangle; and HT-PsJN (PsJN-inoculated plants under high temperature)—red, filled triangles. Trendlines follow similar colors as their corresponding treatments. Points were taken at different time intervals (5, 7, 9, 12, 14, 16, 19, and 21).

The relationship between the number of 1st-order lateral roots and the length of the primary roots where they branched out from showed different correlations (Figure 5a). When we analyzed the fitted line per treatment, the association that followed was an exponential relationship between the two given variables. In general, there was an exponential increase in the number of emerging 1st-order laterals with the growth of the primary roots. The strongest association was found in the control plants at R^2^ = 0.9165, which was followed by the inoculated plants at R^2^ = 0.8688, which were both under ambient conditions. The plants that were subjected to the high temperature showed lower correlations, with that of the control plants being R^2^ = 0.814 and the weakest one from the inoculated plants being R^2^ = 0.3516 (Figure 5a).

A polynomial trendline was the best fit for the relationship between the number of 2nd-order laterals and the corresponding number of 1st-order laterals (Figure 5b). Although they are showing an increase through time, the number of both of the lateral roots did not increase at a constant rate. As with the relationship of the variables in Figure 5a, the highest association was also found in the ambient control plants (R^2^ = 0.8308), while the lowest one was found for the heat-stressed inoculated plants (R^2^ = 0.4925) (Figure 5b). The strong correlation for the different roots of the inoculated plants under the ambient condition (Amb-PsJN) indicates the already established beneficial effects of the bacteria PsJN for the unstressed plants, however, the low correlation that was found in the heat-stressed inoculated plants suggests that there were changes in the dynamics of the growth promotion.

### 2.6. Bacterial Inoculation under Different Temperatures Influences the Leaf Area and Dry Weights with Different Magnitudes

**Figure 6:**
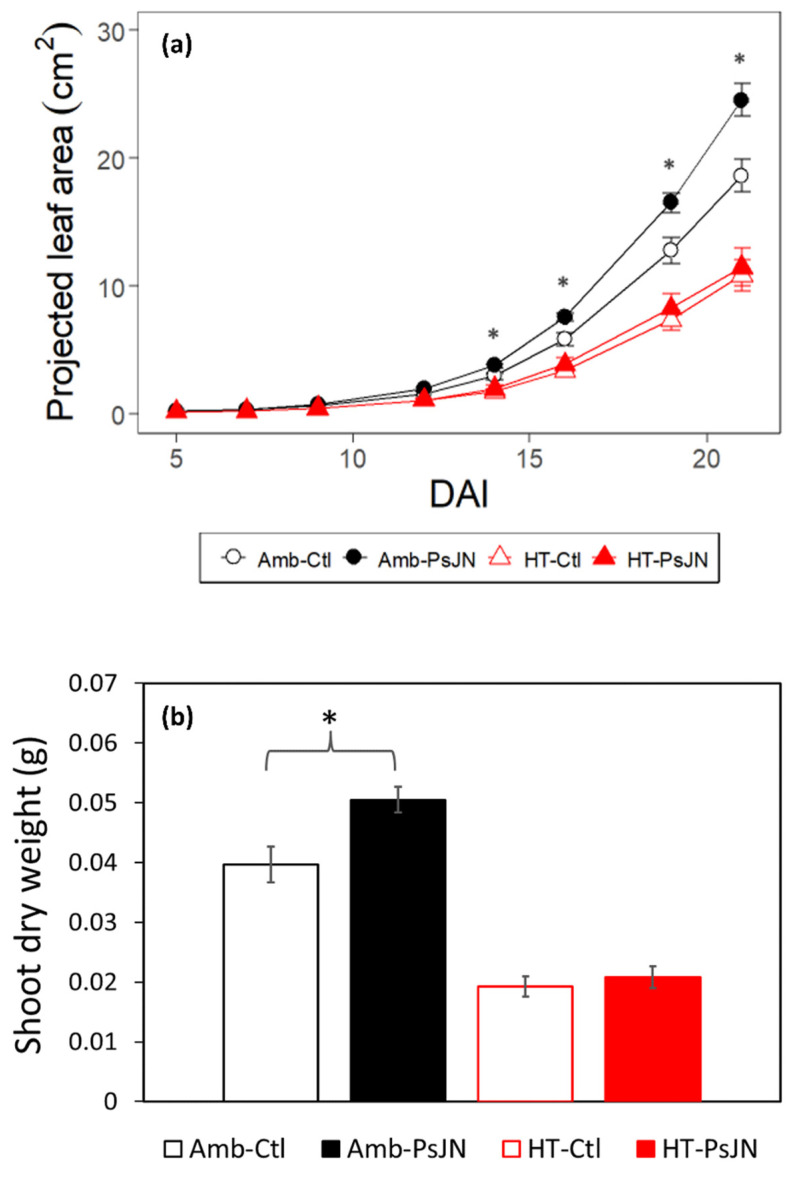
**Projected leaf area and dry weight.** (**a**) Measured projected leaf area of Arabidopsis rosettes growing outside of the agar-filled plates with or without bacteria PsJN inoculation under ambient or high temperature conditions. Temperature—black and circle symbol (ambient temperature), red and triangle symbol (high temperature); bacterial application—empty symbol (control), filled symbol (PsJN-inoculated). (**b**) Dry weights of shoots from all treatments during invasive harvest. Treatments: Amb-Ctl (control plants under ambient temperature), Amb-PsJN (PsJN-inoculated plants under ambient temperature), HT-Ctl (control plants under high temperature), and HT-PsJN (PsJN-inoculated plants under high temperature). All of the points are the mean ± standard error of *n*= 12 (Amb-Ctl), 15 (Amb-PsJN), 12 (HT-Ctl), 8 (HT-PsJN) samples within each treatment. Asterisks: Black—significant difference between mean of PsJN-inoculated and control plants under ambient condition; these are based on the Student’s *t*-test with *p* < 0.05.

The high temperature caused a decrease in the projected leaf area commencing at 14 DAI (Figure 6a). Overall, the high temperature caused a reduction of 51% in the shoot dry weights of the control plants and a 47% reduction in this in the inoculated plants (compared to the ambient control plants) (Figure 6b).

The application of the bacteria positively impacted the projected leaf area under the ambient temperature; however, this effect only significantly manifested from 14 DAI (Figure 6a). This stimulation increased the leaf area by an average of 29%, which is about three times more than it was in the high-temperature counterpart. Comparing the dry weights of the shoots at the time of their harvest, the PsJN-inoculated plants had 0.01 g (25%) higher accumulated dry weights than the control plants did under the ambient condition, whereas only a minimal difference of 0.002 g (12%) was found under the high-temperature condition (Figure 6b).

## 3. Discussion

Our studies utilizing both the conventional closed-plate and a new open-top systems demonstrated the growth stimulatory effects of the *P. phytofirmans* PsJN bacteria on the roots and shoots of the Arabidopsis plants at the ambient and high-temperature conditions. The morphological responses and growth dynamics of the roots and shoots were resolved by monitoring their development through non-destructive phenotyping which was combined with destructive harvesting. Here, we discuss the changes in the root system architecture of Arabidopsis in light of the known regulatory events by intrinsic factors such as hormones, as well as external biotic and abiotic environmental stimuli. Then, we discuss the unique characteristics of the bacterial strain PsJN that stimulated the plant root architectural changes, thus influencing the plants’ overall development and adaptation to the high-temperature stress. We also discuss the advantages of the GrowScreen-Agar II platform over the conventional plant phenotyping pipelines in terms of a strategically designed plant cultivation system, the high-resolution imaging capacity, and the streamlined root and shoot trait quantification.

### 3.1. Changes in the Root System Architecture Are Modulated by Intrinsic Factors and Different Root–Environment Interactions

The interaction of Arabidopsis with the bacteria PsJN and the high temperature altered its RSA. This follows the response of the plant roots to the environment involving the adjustments to the organization of the structural components of the roots from a cellular to a whole-plant level [60]. Arabidopsis has a primary root and several orders of lateral roots (the 1st- and 2nd-order lateral roots, which have been investigated here (Figure 2 and Figure 4)).

The RSA exhibits plasticity under a changing environment, demonstrating the morphological responses in this study. In agreement with Calleja-Cabrera et al. 2020 [18], the high-temperature stress induces several morphological responses in the roots including changes such as the decrease in the primary root length; the reduction in the number, length, and emergence angle of the lateral roots; the increase in the diameter and number of the second and 3rd-order laterals (Figure 2 and Figure 3); the increase in the density of the root hairs, which were not investigated here. Plant roots need an optimal temperature range for them to achieve a proper growth rate and functioning [5,61]. The response of the RSA to increasing temperature can be species-specific since the different plants have different optimum temperature requirements for their growth [62]. An increase in the temperature is associated with the changes in the plant metabolism and nutrient uptake [63]. This uptake is closely associated with the size, morphology, and functioning of the root system [64]. The modifications of the RSA and changes to the root growth which are induced by high temperatures can have undesirable effects on the plant’s ability to capture resources due to the reduced volume of root access [61]. These heat stress-induced alterations to the root growth and structure were found to be due to a reduced rate of cell division [65], as well as the reduced elongation and cell production rate [66]. Changes in the RSA that occurred due to the high temperature also reduced the root-to-shoot ratio [67], linking the belowground to the aboveground biomass allocation patterns and the resource acquisition in plants [68]. This consequence of a modified RSA due to the high temperature was shown in our study for the shoots’ development (Figure 6). It may be that the root trait response to the elevated temperature influenced the carbon fixation and nutrient acquisition, which may have caused the decrease in the shoot trait response [69,70].

PGPR have been described to affect the post-embryonic root development by altering cell division and differentiation within the primary root as well as affecting root hair formation and lateral root development [71]. The most common PGPR-induced root phenotype is either the inhibition of the primary root growth when it is coupled with the proliferation of the lateral roots and root hairs [72], leading to an increased shoot biomass, or the increase in the primary root growth that is coupled with an increase in the plant biomass [73]. The PGP bacteria PsJN that were used in this study had positive effects on the primary, 1st-, and 2nd-order lateral roots, although we also found that the magnitude and direction of the growth stimulation varied depending on the root type and trait, timing, and temperature condition (Figure 2, Figure 3 and Figure 4). Unlike obligate symbionts, PGPR can interact with numerous host plants, modifying the RSA to improve the plant’s health and stress tolerance through a multitude of direct and indirect mechanisms [26,27,28]. These include the formation of biofilms; the production of phytohormones (e.g., jasmonic acid, salicylic acid, gibberellins, indole-3-acetic acid (IAA)), exopolysaccharides, and 1-aminocyclopropane 1-carboxylic acid (ACC) deaminase [74,75]; the production of cell-wall-degrading enzymes, antibiotics, hydrogen cyanide, siderophores and quorum quenching [24,76]. These PGPR-induced modifications of the RSA allow for the enhanced functions of different root regions, which are responsible for soil exploration for nutrient acquisition [77]. The PsJN bacteria that were used in this study also stimulated the increase in the projected leaf area, and this, together with the root growth promotion, may have led to a higher shoot dry weight, the magnitude of which varied according to the temperature condition (Figure 6). The simultaneous capture of these time-specific events under the bacterial application in two temperature regimes on both the root and shoot growth dynamics are some of the new findings of this study which have not been previously reported.

### 3.2. P. phytofirmans PsJN Induce Modifications of the Components of the Root System Architecture Contributing to Improved Plant Growth

The magnitude of the bacteria-imparted growth promotion on the root architecture varied depending on the specific root type, which to our knowledge, has not been dissected before, and was influenced by the temperature and time. Significant effects due to the bacterial inoculation were observed in the root lengths, growth rates, and the number of the lateral roots (Figure 2a–g,i,j). These results support other studies showing that PGPR assist plants by facilitating the elongation of the root tissues and improving their growth rates for the optimized absorption of available nutrients [32,78]. The increase in the depth of the roots indicates the prominent effect of the bacteria PsJN on the primary roots (Figure 2b,g). This can be attributed to the bacteria’s involvement in the main processes governing cell division and cell elongation, which are mainly regulated by the phytohormones [79,80], ACC deaminase, siderophores, and other secondary metabolites [39,81], which have been proposed to act as signaling molecules during plant root–bacteria communication for efficient root colonization [82]. On the other hand, the increased formation and elongation of the lateral roots (Figure 2c,d,h,i) that underwent bacterial inoculation may also be due to the alteration of the endogenous pool of growth-regulating hormones such as IAA by the bacteria PsJN [1,39].

This study also showed significant increases in the number of lateral roots that underwent bacterial inoculation (Figure 2e,j). This coincides with the outcome of in vitro potato plantlets that were inoculated with a PsJN strain showing a better performance in the plants’ growth, likely due to the development of more secondary roots [83], although our study has further dissected the contribution of the 2nd-order lateral roots (also known as tertiary roots). The timing and location of the emergence of these lateral roots also contributed to the density and distribution of the roots across different depths and to means by which the application of the bacteria affects the total root lengths (Figure 4a–c). The increase in the root length distribution towards the deeper or distal part of the root system by the bacteria PsJN indicates the potential for more water and nutrient foraging when the upper edaphic environment has become unfavorable for the roots. When they were compared to the primary roots that traced their origin back from embryogenesis, the lateral roots form post-embryonically, allowing for the dynamic acclimation of the whole root system architecture to environmental fluctuations over time [52,84]. The strong correlation between the number of 1st-order laterals against the increase in both the length of the primary roots and the number of 2nd-order laterals (Figure 5a,b) that underwent inoculation indicates the stimulatory effect of the PsJN bacteria on the growth of these roots at an ambient condition. This suggests one of PsJN’s adaptation benefits to the plants. The increase in the number of lateral roots does not only widen the root scavenging area [85], but this also provides a crucial adaptive response to elevated temperatures, which is generally accompanied by a reduced availability of water and nutrients in field settings. Early vigor of these root types can provide an advantage for seedling establishment in preparation for the later growth stages and environmental challenges [86].

### 3.3. Positive Bacteria-Imparted Root Modifications Correlate with Shoot Responses at Ambient Temperature and Are Time-Dependent

The positive effects of PsJN on the morphology and growth dynamics of the individual root types manifested early during the root development, before declining after certain time points (Figure 4). This trend of initial bacterial stimulation and decline was exhibited mainly by the primary and 1st-order lateral roots. On the other hand, the 2nd-order lateral roots showed a continuous increase from the moment that the inoculation was performed, which can be ascribed to the late development of these root types. Contrary to the early growth promotion that was imparted by PsJN in the roots, which started at 5 DAI, the enhancement of the rosette surface area of the shoot came 14 days later (Figure 6a) under the ambient conditions. An extended time-series measurement might be needed to fully elucidate the potential of the bacteria PsJN for both the root (2nd-order laterals) and shoot improvement as our growth system was limited by the size of the plates.

Whilst they can be found in the aboveground tissues of the plants, the rhizosphere bacteria *P. phytofirmans* PsJN generally reside inside the root tissues, although some are also located in the rhizoplane [87]. Since the plants usually first invest in root growth at the early establishment stage [63], the growth promotion that is imparted by the PGPR strain PsJN may also be reflected in these growing root components. The late-stage significant enhancement of the rosette surface area by the bacteria may just be following this growth trend. It may also be that the plant growth promotion in the shoot is not (only) through the increase in the rosette surface area, but it is also from other shoot traits such as the growth rates and the number of rosette leaves [46]. The results of measurements of the shoot dry weights at the point of harvesting them (21 DAI) indicate that there was a strong bacterial stimulation effect, with the inoculated plants being about 25% heavier than the control plants were under the ambient temperature (Figure 6b). This observed increase in the shoot dry matter could be explained by the stimulation of the primary root growth and root branching [83], which consequently lead to better nutrient and water uptake. This may indicate that the benefit of using PsJN will potentially transfer to the shoot biomass, and it will be useful for agriculture.

### 3.4. The Extent of Bacterial Stimulation Effect in Roots and Shoots Varies Depending on the Temperature Condition

The modulation of the growth that was induced by PsJN on Arabidopsis varied in intensity between the two temperatures that were investigated in our study. Although the beneficial effects of the bacteria were significant under the ambient condition on the root tissues, these effects were more substantial, with there being higher relative differences between the inoculated and non-inoculated types under the heat-stress conditions (Figure 2). For example, at the end of the growing period, the difference in the root lengths of the inoculated and non-inoculated plants under the heat stress condition based on individual root parts was about 3.5 times higher than it was in the primary roots, 3.8 times higher in the 1st-order laterals, and about higher 2.1 times in the 2nd-order laterals than it was in the ambient condition. The strain PsJN is already known for its plant-growth promotion benefits under normal conditions; however, plants can take even more advantage of this bacteria’s imparted traits under stressful high-temperature situations when the impacts can be quite deleterious to the plants. For example, in potatoes, a high temperature caused a reduction in the root and shoot development, the number of potato tubers, and the fresh weight of them, however, the PsJN inoculation showed noticeable beneficial effects on the root biomass, and significantly enhanced the number of tubers and the weight of some cultivars [35].

The thermotolerant capability that was imparted by the bacteria might be attributed to its production of ACC deaminase, which has been linked with the alleviation of plant stress because of its contribution in lowering the ethylene levels, thereby promoting plant growth [37,88]. ACC deaminase-producing bacteria enhance the plants’ growth under different biotic and abiotic environmental conditions such as pathogen attacks, drought, salinity, and organic and inorganic contaminants [1,89]. Furthermore, microbial deaminases have been shown (or proposed) to be responsible for the dissociation of stress-induced ACC (secreted as root exudates) into ammonia and α-ketobutyrate, thereby eliminating ACC, the precursor for ethylene that has a drastic impact on the physiology, growth, and development of plants [90,91,92]. Under periods of stresses, ethylene can facilitate the early senescence and abscission of various organs, which are mechanisms to conserve resources [93,94]. The role of microbial ACC deaminase producers such as PsJN might be immensely important in today’s agricultural systems, where stressed-induced ethylene changes in crops are aggravated by increasing temperature conditions.

Although the PGP effects of the bacteria were strongly demonstrated on several root parameters such as the root lengths and the growth rates (total, primary, and 1st- and 2nd-order laterals), as well as the number of 1st- and 2nd-order lateral roots, these results did not translate in the root system traits (Figure 3a–d). The conditions in agar where the water and nutrients are homogenized are different from they are in the soil where there is a need to expand the volume that is covered by the roots as there will be a very likely heterogenous distribution of the edaphic resources. Still, we could show that PsJN causes spatial distribution effects (Figure S3), and that the bacterial enhancements for improved nutrient uptake were manifested in the individual root component, e.g., the longer lateral roots (Figure 2), and in the changed root length distribution, e.g., an increased root density at the deeper part the roots (Figure 4).

### 3.5. Temporal Effects of Bacterial Inoculation Show Accelerated Growth Rates at the Early Stage of Plant Growth Depending on Root Type and Temperature Condition

Although the growth stimulation imparted by the bacteria was a prominent trend, the difference between the inoculated and the control ones varied at different stages of the plants’ development—initially increasing and then decreasing at certain timepoints. Under the ambient condition, the effect of the bacterial inoculation on the root lengths and growth rates showed early in the root development, and the stimulation effect increased up to a certain timepoint depending on the root type (Figure 2b–d,g–i). For example, the stimulation on the primary roots increased up until 9 DAI and until 12 DAI for the 1st-order lateral roots, while the 2nd-order laterals showed continuous growth and bacterial stimulation until 21 DAI. After these time points (except for the 2nd lateral roots), the stimulation effect declined although there were still significant differences between the inoculated and non-inoculated plants.

This outcome, in part, corroborates the result that was found by Poupin et al. 2013 [46]. They found that drastic changes occurred, including accelerated plant growth in Arabidopsis in their inoculation study at 13 days after sowing them. However, although the strain PsJN accelerated the growth rate during the first half of the plants’ development, the growth rates then levelled off and the plant size converged with that of the non-inoculated plants. In our study, it may be as well that if the plants were allowed to grow further, a similar outcome might have taken place. For the shoots, their study demonstrated that the larger rosette areas of the PsJN-inoculated plants during the first half of their life cycles are correlated with the larger leaf areas, rather than there being an increase in the number of leaves. This illustrated that the bacteria PsJN acted as PGPR by accelerating the growth rates, and thus producing bigger plants [46]. The accelerated growth in the early stages of the inoculated plants, which can be attributed to the various mechanistic effects of the bacteria, may provide beneficial effects to the plants including improved nutrient acquisition and/or a direct effect on the plant metabolism [46]. On the other hand, based on the living theory that longevity is negatively correlated with the metabolic rate [95], the rapid growth that occurred during the early stages of life may be associated with a reduced longevity and an impaired future performance [96]. This may be the case with heat-stressed plants, where the accelerated growth rates, particularly during the early growth stage, are generally followed by an earlier reproductive and flowering stage.

### 3.6. The GrowScreen-Agar II Is an Efficient Platform for Plant Cultivation, Non-Invasive Phenotyping, and Root and Shoot Trait Characterization

The GrowScreen-Agar II platform provided an efficient growing system that enabled a more natural growth to take place for the Arabidopsis roots and shoots as compared to the conventional “closed-plate” setup. The previous version of this platform was compared to the already existing phenotyping setups in [52]. Notably, the strategic design of the agar plates allowed the unobstructed, open-air growth of the shoots which still is a distinguishing feature, also, in the newer systems. This is also a trait in another recent platform GLO-Roots [97,98] which has a similar “open-top” approach, while the MultipleXLab [99] one sticks to the conventional “closed-plate” system. Having access to the leaves opens the potential for the non-invasive monitoring of the plant’s physiological parameters such as chlorophyll fluorescence and gas exchange, i.e., net photosynthetic rate, stomatal conductance, and transpiration, which are important indicators of the plant’s response to the climate and environmental changes (e.g., [4,100,101]). In this study, however, the small size and orientation of the Arabidopsis rosettes (Figure 1a,b) restricted the use of gas exchange measuring instruments, which should not be a problem with the other species. Aside from the shoot benefits, this platform explicitly addresses the problems of growing and phenotyping roots that are grown in the dark, which is also accounted for in GLO-Roots, but is still an issue in the setups like MultipleXLab. Moreover, a more detailed and periodic investigation of the different root traits pertaining to the root system architecture was also made possible. Although this platform is still to be fully automated, it has successfully provided a multi-functional imaging system for non-invasive phenotyping and the in-depth characterization of the roots and shoots up to a period of 21 days by extending the plates. GLO-Roots similarly imaged the Arabidopsis roots up to 31 days in even longer containers, whereas the automated microscope MultipleXLab only focused on the seedlings in the first 4 days. In addition, this phenotyping system made it possible to grow plants that were subjected to external environmental conditions, such as the addition of the PGPR strain PsJN and the control of the temperature. Furthermore, GrowScreen-Agar does not require plant transformation or watering with luminescent reagents like in GLO-Roots, but it can handle plants of any type of background (as can the light-based imaging of MultipleXLab). This also has the potential for investigating more than one gnotobiotic microorganism under the combined variable abiotic treatments, which is the direction of the plant-microbe interaction studies under future climate challenges [102,103]. This platform’s associated image analysis software demonstrated the reliable quantification of the morphological and dynamic responses of the root and shoot traits against a combined bacteria and high temperature condition.

Once it is completely functional, the GrowScreen-Agar II platform will be a promising solution for high-throughput plant cultivation and non-invasive phenotyping within a fully automated system. However, despite the improvement in the available root space for this study, the platform is still constrained. A limitation of the agar-based plant cultivation method (both GrowScreen-Agar, MultipleXLab and others) is the artificial environment that is uses which differs greatly from the natural niche of the plants and microorganisms in the soil or the field. here, GLO-Roots that uses soil as a growth medium has a slight advantage with the compromise that soil often obscures root imaging which could prove challenging at the branching points in complex root systems. For all of the three platforms (similar to findings from all of the artificial systems), it remains true that while they capture various aspects of the plants’ response mechanisms and quantify the changes in them, these findings need to be further validated in realistic environments where plants have a larger soil area to scavenge and are exposed to all of the biotic and abiotic elements.

## 4. Materials and Methods

### 4.1. Customized Agar-Plate Preparation

The GrowScreen-Agar II platform utilizes specifically designed plates that were developed in collaboration with and manufactured by the company Happ Kunststoffspritzgusswerk und Formenbau GmbH (Ruppichteroth, Germany). The plates which were manufactured by injection molding, which rendered them sterile for our use, are comprised of three components: an opaque cover, a transparent back plate with holes on top, and a black top part (“collar”) (Figure S4). The opaque cover consists of polypropylene (PP) and is equipped with an anti-fog agent to prevent water droplets that would disturb root image analysis. The transparent back plate consists of polystyrene (PS) and allows non-invasive imaging of roots growing in the agar. The shoot growth outside of the plate is enabled through 3 holes with a diameter of 5 mm on the short side of the transparent plate (the distance between the holes is 29 mm with one hole exactly in the middle of the short side). The black collar (polypropylene with 30% glass fiber, PP-GF30) had three holes in line with the holes in the transparent back plate, but with a reduced diameter of 2 mm. Its primary function was to keep as much light out of the root part of the plate and to provide a proper background for shoot imaging (Figure S4).

Before seed preparation, the holes on the back plates were sealed using micropore tape before being filled with modified Hoagland and agar media (composition in a liter of Milli-Q water: 1.67 mL KNO_3_, 1.67 mL Ca(NO_3_)_2_∙4H_2_O, 0.67 mL MgSO_4_∙7H_2_O, 0.33 mL KH_2_PO_4_; 0.33 mL of trace elements (MnCl_2_∙4H_2_O, CuSO_4_∙5H_2_O, ZnSO_4_∙7H_2_O, H_3_BO_3_, Na_2_MoO_4_∙2H_2_O); 0.33 mL [Fe^3+^-EDTA]^−^; and 1% Agar) [52]. The media were autoclaved and poured into the plates (approximately 225 mL capacity) inside the biosafety cabinet. Once the agar cooled down and solidified, the opaque covers and collars were attached, and the assembled plates were stacked and sealed in their original bags to maintain sterility before sowing.

### 4.2. Seed Sterilization, Sowing, and Stratification

*Arabidopsis thaliana* Col-0 (hereafter referred to as Arabidopsis) seeds were surface sterilized using 70% (*v/v*) ethanol solution and 0.5% (*v/v*) sodium hypochlorite solution with 0.05% (*v/v*) Tween 20 (VWR International GmbH, Darmstadt, Germany) (5 µL per 10 mL solution). In brief and operating in a biosafety cabinet, Arabidopsis seeds in 2 mL Eppendorf tubes were first incubated with 0.5 mL 70% ethanol for three minutes. After incubation, ethanol was pipetted out and replaced with 0.5 mL of 0.5% sodium hypochlorite solution. The tube was slowly mixed while it was incubated for 10 min. The solution was then pipetted out, and the seeds were washed with autoclaved Milli-Q water three times. After washing, the seeds were suspended in 0.5 mL of autoclaved Milli-Q water and set aside for sowing.

To prevent any contamination, sowing was performed inside the biosafety cabinet. Individual Arabidopsis seeds were sown into the pre-prepared agar-filled customized plates. A single seed was slowly pipetted out from the sterilized batch and carefully dispensed into a hole on the plate. When necessary, a sterile syringe needle was used to position the seed into the middle of the hole. When all of the holes were sown with seeds, the collars were reapplied then sealed with parafilm, after which, the plates were bagged aseptically and placed horizontally in the fridge at 4 °C for five days. Subjecting Arabidopsis seeds to a period of cold temperature breaks dormancy and softens their coat, allowing for uniform germination. At the end of the stratification period, the plates were transferred back into the sterile bench and the parafilm was removed for bacterial inoculation.

### 4.3. Bacterial Cultivation and Inoculation

Paraburkholderia phytofirmans PsJN (DSMZ, Germany) (here, referred to as PsJN) was routinely grown on Luria-Bertani (LB) media (1.2% agar *w/v* with no salt). A day before inoculation, a single bacterial colony from an agar plate was aliquoted into an LB broth, and the bacterial solution was cultured overnight in an orbital shaker (150 rpm) at 30 °C. The cell suspension was measured for optical density (OD600) using a portable spectrophotometer and when an OD600 value of 0.8 (equivalent to 108 colony-forming unit (CFU) mL^−1^) was reached, cells were centrifuged down, washed, and serially diluted with Hoagland media to obtain a concentration of 104 CFU mL^−1^. A study by Poupin et al. 2013 [46] reported this bacterial concentration as the optimum for plant-growth promotion in Arabidopsis. Inoculation was performed by pipetting 10 µL of either PsJN-bacterial inoculum (inoculated) or Hoagland solution as mock inoculant (control) onto each seed. After all of the seeds received appropriate treatments, the black collars with seed-sown holes were sealed with parafilm, and the inoculated and control plates were transferred to separate growth chambers with varying temperatures.

### 4.4. Plant Growth Conditions

The number of plates was divided into two groups corresponding to ambient and heat stress conditions on separate growth chambers with 22/18 °C and 30/24 °C day/night temperatures, respectively. In both climate chambers, the plants were grown under a long day period of 16/8 h, receiving 120 µmol/m^−2^ s^−1^ of light and 60% relative humidity. The plates were positioned vertically and maintained in fabricated metal magazines (660 × 210 × 129 mm) fitted for the plates (10 plates per magazine) (Figure S5). This open-top system allowed for the unobstructed, upward, open-air growth of the shoots, whilst simulating the downward growth of the roots in the dark. During the first seven days, the holes remained sealed with parafilm to provide a high-humidity condition during germination and early plant development. After this period, the parafilm was removed and, if needed, a syringe needle and/or small tweezer were used to ensure the shoots were growing outside the holes (Figure 1a). The plants were cultivated for 21 days after inoculation (DAI), with routine imagining and phenotyping using the GrowScreen-Agar II imaging system.

### 4.5. GrowScreen-Agar II Platform Specifications

The GrowScreen-Agar II system has been modified from the original GrowScreen-Agar I system [52]. GrowScreen-Agar I was designed for imaging traditional square Petri dishes (127 × 127 × 17 mm), having both a top camera for the shoot and a camera with a white backlight for root imaging. The GrowScreen-Agar II was designed for longer (taller) plates (plate: 200 × 100 × 19 mm, collar: 136 × 60 mm), and it was equipped with three cameras for imaging: one for the root (29 MPx Prosilica GT6600, Allied Vision Technologies GmbH, Stadtroda, Germany) and two cameras (top and side view) for the shoot (5.1 MPx Mako G-507C, AVT GmbH, as above). Root illumination was achieved from the back using an 850 nm infrared LED panel (EFFI-BL-150T-250-850, EFFILUX, Les Ulis, France), and therefore, also, an IR-capable lens was used (Xenon Emerald 50/2,2-F-S, Jos. Schneider Optische Werke GmbH, Bad Kreuznach, Germany). Shoot cameras were equipped with white LED rings (LDR2-70-SW2, CCS Inc., Kyoto, Japan) and standard lenses (LM8JC3M, Kowa Optronics Co., Ltd., Nagoya, Japan and Xenoplan 1.9/3, Jos. Schneider Optische Werke GmbH, Bad Kreuznach, Germany). This imaging system (Figure S6) was strategically positioned inside the growth chamber. In this study, the plates with growing plants were manually transferred into the imaging station. The imaging was driven by a custom software program that was implemented using the Software Development Kit (SDK) NET-based OPC UA Client Server SDK Bundle (Unified Automation GmbH, 90562 Kalchreuth, Germany). After imaging, the plates were placed back into their respective growth temperature chambers (VB 1014, Vötsch Industrietechnik GmbH, 72336 Balingen-Frommern, Germany).

### 4.6. Growth Optimization Using a Traditional “Closed-Plate” System

Before using the “open-top” system, the effects of PsJN were initially investigated in a small experiment using the conventional “closed-plate” set-up. This was used to determine the optimal growth condition for both plant and bacteria, the time-series effect of the inoculation with the corresponding root growth timepoint, and the morphologic responses of the roots to bacteria under ambient and heat stress conditions. In this system, Arabidopsis seeds which had been sown on the surface of agar plates were inoculated with the bacteria PsJN two days after germination when the radicles were about 2–5 mm. The plates were sealed with micropore, placed in a vertical position, and divided into two growth chambers with ambient and heat stress settings (similar to the abovementioned growth chamber settings). At several timepoints, the plates were removed from the growth chamber and scanned using WinRhizo. Initial analysis was performed using the WinRHIZO™ 2019 software.

### 4.7. Time-Course Image Analysis

The plates were transferred to the imaging station of the GrowScreen-Agar II and imaged non-destructively at the following time intervals: 5, 7, 9, 12, 14, 16, 19, and 21 days after inoculation, thus producing the shoot and root images (Figure 1b).

Shoot analysis: The shoot images (.tiff files) which were captured using the top camera were analyzed with a color space-based segmentation approach to compute the projected leaf area as a proxy for the rosette surface. We used software from a toolbox to estimate leaf angles from stereo images [104] with the revised version of the segmentation tool that was published separately in [59]. Images were pre-processed to remove chromatic aberrations which appeared between contrasting regions. In the filtered images, plant pixels were identified by thresholding operations in the HSV color space [104]. If it is not stated by us, upper thresholds were set to maximum. Lower and upper hue channel (0–360°) thresholds were set to 21° and 222°, and lower thresholds in the saturation and value channels (0–1) were set to 0.11 and 0.17, respectively. The last two dates (19 and 21) were processed with a value channel threshold of 0.08. Two post-processing filters were applied to remove smaller pixel clusters (a group of continuous pixels of category plant or background) resulting from misclassifications in the background and the plants. Pixel clusters of the plant category exceeding a size of 95 pixels were converted to background pixels, while background pixel clusters exceeding a size of 24 pixels were converted to plant pixels. For the last two dates, we increased these thresholds to 502 and 473, respectively. The result is a semantic segmentation with white plant pixels and black background pixels. The projected leaf area was computed from the sum of white pixels, which was converted to metric values (Figure 1c (top)).

Root analysis: The root images (.tiff files) were first arranged, processed, and then analyzed using the image analysis software GrowScreen-Root [52,105]. The software allows us to distinguish between the different root types: the primary roots (green), 1st-order lateral roots (red), and 2nd-order lateral roots (blue). GrowScreen-Root also measures the following root traits: root lengths (primary, 1st-, and 2nd-order laterals, and total), convex hull area, root system depth, root system width, number of lateral roots, as well as the branching angle of the laterals (for trait descriptions, see [52] (Figure 1c (bottom)).

### 4.8. End-Point Harvest and Validation of Bacterial Colonization

At 21 DAI, plates were removed from the growth chambers and the plants were harvested. Plates with root tissues that were allocated for the determination of bacterial colonization were placed inside the biosafety cabinet. The remaining plates were used for shoot harvesting.

Since the PsJN bacterial inoculum was added to the seeds on the open surface of agar plates, we tested whether the bacteria travelled with the downward growth of the roots inside the agar and whether the inoculated samples were colonized by the bacteria at the level of root tips. For rhizoplane colonization, 1–2 cm of the root tip was cut and placed in a 2-mL Eppendorf tube with LB media, washed, vortexed, and the resulting washing was serially diluted and plated. For endophytic colonization, the washed root tissue from the first tube was placed in a separate clean Eppendorf tube where it was first macerated before being resuspended with LB broth. An aliquot of the resulting mixture was then serially diluted and plated. These bacterial colonization tests were performed on both PsJN-inoculated and control roots to check for unwanted microbial contaminations (Figure S2). From the plated cultures of the bacteria, a single colony was used for PCR of the 16S rRNA bacterial gene using the universal bacterial primers 27F (5′-AGAGTTTGATCMTGGCTCAG-3′) and 1492R (5′-TACGGYTACCTTGTTACGACTT-3′). Part of the PCR product was cleaned up and sent for sequencing to check if the sequence matched to PsJN (Figure S2c).

### 4.9. Statistical Analysis

The statistical difference of measured parameters between the two treatments and the effect of microbe application under ambient or heat stress conditions were analyzed using the Student’s *t*-test (two-tailed distribution). In addition, overall treatment effects such as microbial application (microbe), heat stress effect (temperature), and interaction effect per individual day and over the entire growing period were also analyzed using the ANOVA function in R studio. Only those results with a significant *p*-value level of <0.05 were considered reliable enough to reject the null hypothesis that state that the two treatments did not differ for a particular parameter.

## 5. Conclusions and Future Perspectives

Our study shows that the bacterial endophyte strain *P. phytofirmans* PsJN improved the shoot and root growth under normal temperature conditions. Depending on the plant tissue and specific trait, this growth enhancement may be more substantial under the high-temperature conditions, thus showing a potential advantage for the use of bacterized plants in future climate change scenarios. We have also demonstrated that depending on the root type and their time of emergence, the stimulation effect that was imparted by the bacteria may be stronger at the early stages of the plant’s growth and then, it may level off towards the end of plant’s development. This observation may allow young plants to grow well-established roots with more access to water and nutrients, which can assist in the later growth stages wherein they are sensitive to environmental fluctuations and challenges. Information about the spatial and temporal effects of this bacteria, particularly in the roots, can inform plant growers about alternative solutions for increasing their crop productivity and the potential mitigation and adaptive strategies in addressing the climate-related impacts on agriculture. Finally, we have demonstrated, here, the potential of the platform GrowScreen-Agar II in providing a more natural plant growth conditions, non-invasive imaging and phenotyping, and trait quantification capabilities for studying the plants’ morphologic responses and growth dynamics.

Crop plant selection is currently being focused on the RSA because of its overarching importance in the plant’s ability to acquire edaphic resources, which is limited by suboptimal water and nutrient availability and exacerbated by climate change [106]. Aside from the genetic improvement and conventional breeding strategies for improving the RSA, the application of PGPR can be an alternative avenue for developing more productive, resilient, and climate-smart crops. This can be extended to using not just a single, but a combination of known beneficial microorganisms. However, further studies on the mechanisms of growth promotion and biotic and abiotic stress tolerance need to be explored to maximize the potential of microbial applications to agriculture. In addition, novel growth systems addressing existing plant cultivation limitations and non-invasive high-resolution phenotyping platforms can also be taken advantage of to understand the underlying biochemical mechanisms behind the plant–microbe–environment interactions for future sustainable biotechnological solutions.

## Data Availability

The data which are presented in this study are available within the article and linked supplementary files.

## Supplementary Materials

The following supporting information can be downloaded at: https://www.mdpi.com/article/10.3390/plants11212927/s1, Figure S1: WinRhizo analyzed root lengths and root and shoot biomass; Figure S2: Root sampling and bacterial colonization confirmation; Figure S3: Sample root images generated by the GrowScreen-Agar II; Figure S4: Agar plates for GrowScreen-Agar II; Figure S5: Magazines for GrowScreen-Agar II; Figure S6: Imaging station of GrowScreen-Agar II; Table S1: Mean values and standard error of different root type morphological traits; Table S2: Mean values and standard error of different root system traits describing distribution and spread.
