# Supplementary material for: Plant Growth Promotion and Heat Stress Amelioration in Arabidopsis Inoculated with Paraburkholderia phytofirmans PsJN Rhizobacteria Quantified with the GrowScreen-Agar II Phenotyping Platform
Source: Plants (Basel). 2022 Oct 30;11(21):2927. doi: 10.3390/plants11212927 (PMC9655538; doi:10.3390/plants11212927)
Supplement: Supplementary file 1 [file plants-11-02927-s001.zip › plants-1974083-supplementary.pdf]

Electronic Supplementary Files

Plant Growth Promotion and Heat Stress Amelioration in Arabidopsis Inoculated with *Paraburkholderia phytofirmans* PsJN Rhizobacteria Quantified with the GrowScreen-Agar II Phenotyping Platform

Allene Macabuhay [1,2,3], Borjana Arsova [2,*], Michelle Watt [1], Kerstin A. Nagel [2], Henning Lenz [2], Alexander Putz [2], Sascha Adels [2], Mark Müller-Linow [2], Jana Kelm [2], Alexander A. T. Johnson [1], Robert Walker [1], Gabriel Schaaf [3] and Ute Roessner [1,4]

[1] School of BioSciences, University of Melbourne, Parkville, VIC 3010, Australia
[2] Institute for Bio-& Geosciences (IBG-2), Plant Sciences, Forschungszentrum Juelich GmbH, 52425 Juelich, Germany
[3] Institute of Crop Science and Resource Conservation, Department of Plant Nutrition, University of Bonn, 53115 Bonn, Germany
[4] Research School of Biology, The Australian National University, Acton, ACT 2601, Australia
* Correspondence: b.arsova@fz-juelich.de

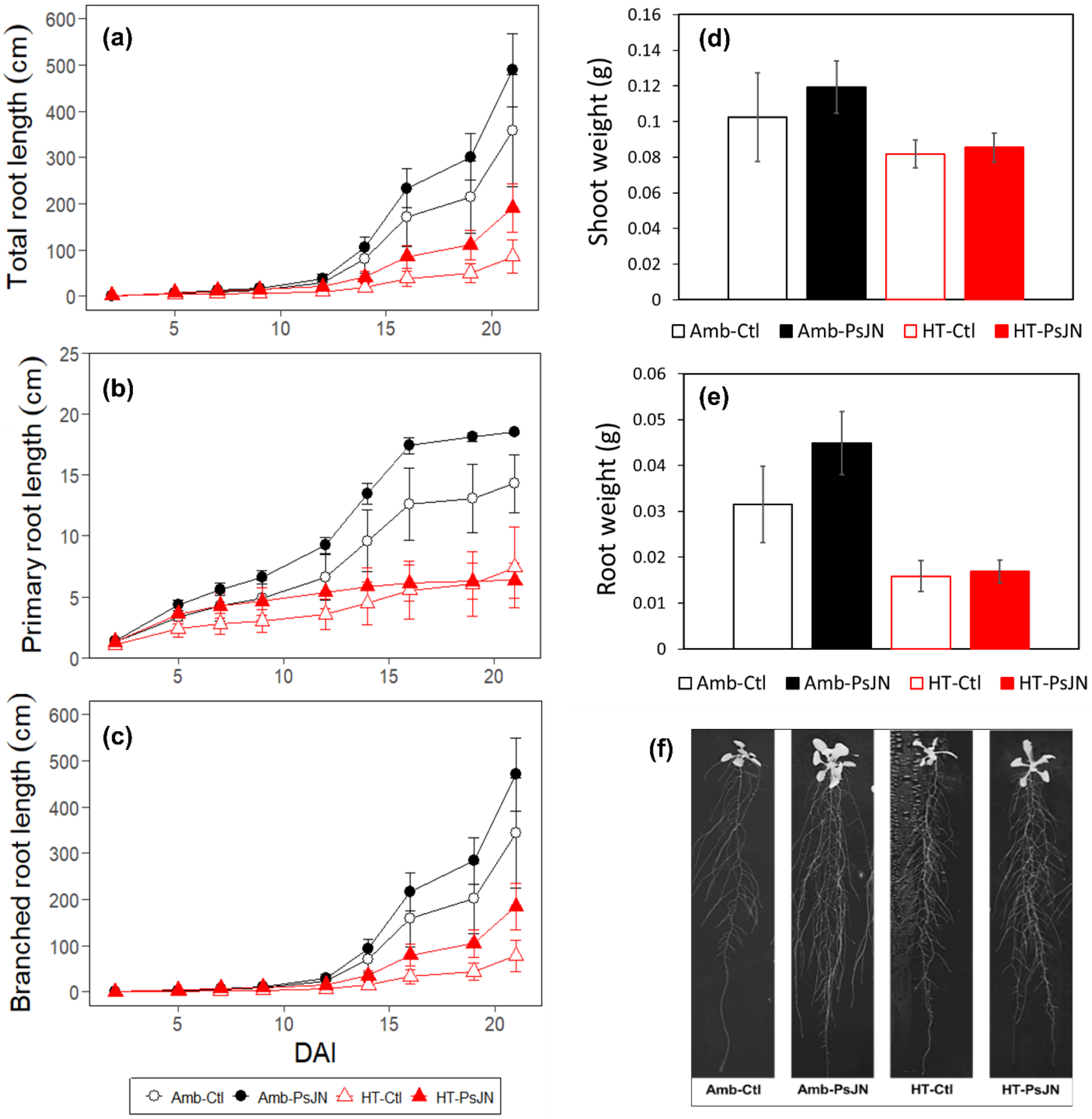

Figure S1. WinRhizo analysed root lengths and root and shoot biomass

Root lengths quantified using WinRhizo analysis in a closed-plate experiment: (a) Total root lengths, (b) primary root length, and (c) branched root lengths. Temperature - black and circle symbol (ambient), red and triangle symbol (high temperature); bacterial application – empty symbol (control), filled symbol (PsJN-inoculated). Plant biomass taken at harvest: (d) shoot dry weight and (e) root dry weight. (f) Sample images of 16-day old seedlings from each of the four treatments. Treatments**:** Amb-Ctl (control plants under ambient), Amb-PsJN (PsJN-inoculated plants under ambient), HT-Ctl (control plants under high temperature), and HT-PsJN (PsJN-inoculated plants under high temperature). All points are the mean ± standard error of n= 6 samples within each treatment. Asterisks: Black –

significant difference between mean of PsJN-inoculated and control plants under ambient condition, red - significant difference between mean of PsJN-inoculated and control plants under high temperature condition, based on the Student's t-test with p<0.05.

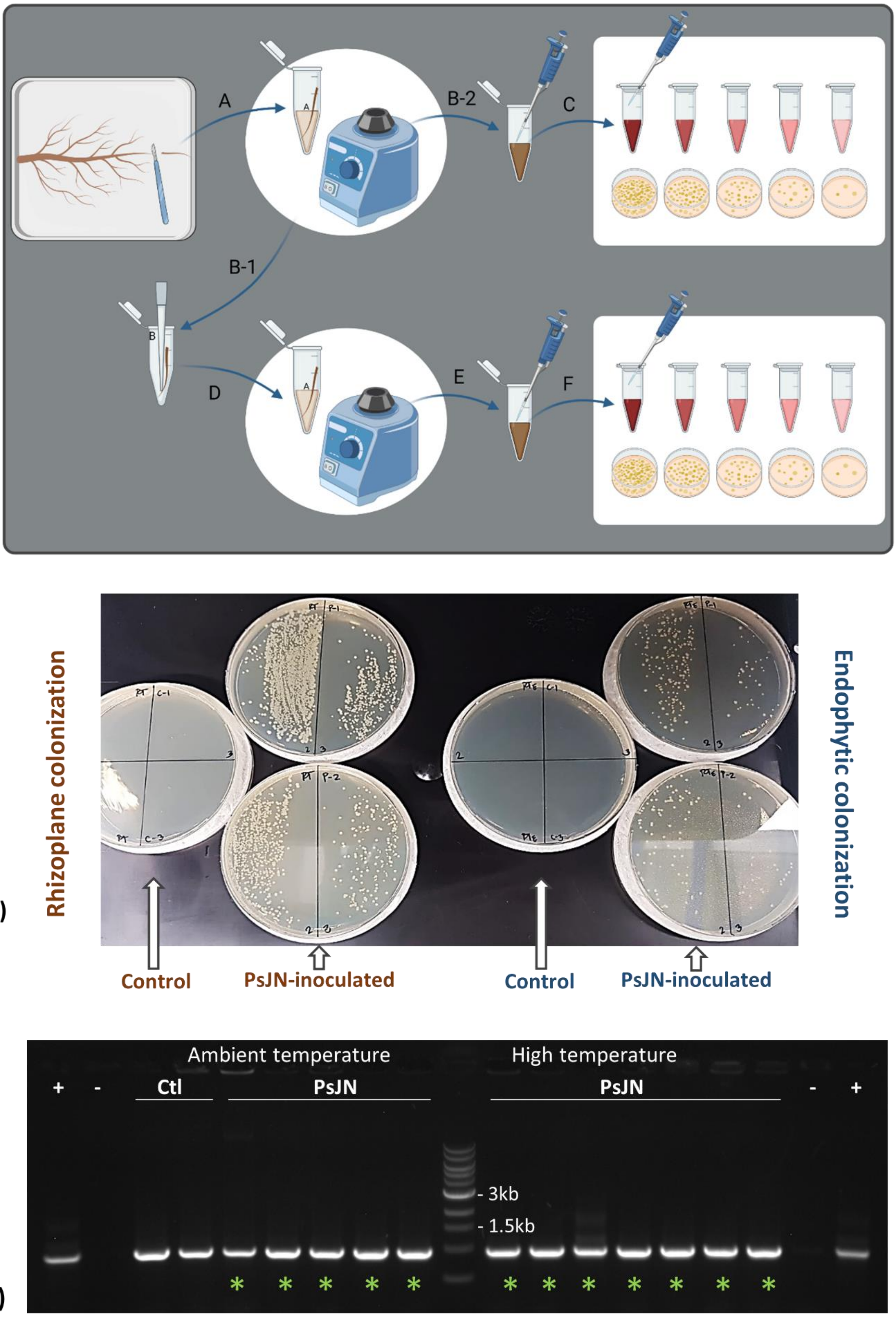
A
B-2
C
B-1
D
E
F
Rhizoplane colonization
Endophytic colonization
Control
PsJN-inoculated
Control
PsJN-inoculated
Ambient temperature
High temperature
+
-
Ctl
PsJN
PsJN
-
+
3kb
1.5kb

(a)

(b)

(c)

Figure S2. Root sampling and bacterial colonization confirmation

(a) Brief procedure for sampling of root tissues for the determination of bacterial colonization. (A) Cutting about 1-2 cm of the root tip, placing into pre-prepared Eppendorf tube with LB (no NaCl) media, and washing/agitating the root using a vortex to get the root surface bacteria. (B-2) Aliquoting from the LB surface washing fraction. (C) Performing serial dilution of the bacterial inoculum, aliquoting from each dilution, and plating. (B-1) Removal of the washed root and transfer into a separate clean tube for maceration. (D) Adding LB media (no NaCl) to the macerated root and mixing using a vortex to extract the bacteria inside the root tissue. (E) Aliquoting from the washing to transfer to tubes for serial dilution and then plating each of the dilutions (F). (C) corresponds to **rhizoplane** colonization while (F) yields **endophytic** colonization of bacteria (image created with BioRender.com). **(b)** Sample plated dilutions at 21 DAI. Left two columns show rhizoplane colonization and right two columns show endophytic colonization of the bacteria. 1st and 3rd columns are from non-inoculated roots showing no growth, indicating sterility of system; while 2nd and 4th are from inoculated roots, showing growth from the bacterial strain PsJN. **(c)** PCR of single bacterial colonies from macerated root tips which have been plated out on LB media. The PCR products were sequenced, and the ones identified as *Parabulkoholderia* sp. are indicated with a green asterix "*". Minimum of 3 plants of each treatment were sampled. Note that the three high temperature control roots and one ambient temperature control root showed no colonies at all on the plate - thus no PCR products, whereas the products in the 2 ambient temperature controls was identified as a *Paenibacillus sp*. "+"- positive control for the universal bacterial primers, "-" – negative control for the PCR reaction, Ctl- control, PsJN- Roots inoculated with PsJN bacteria.

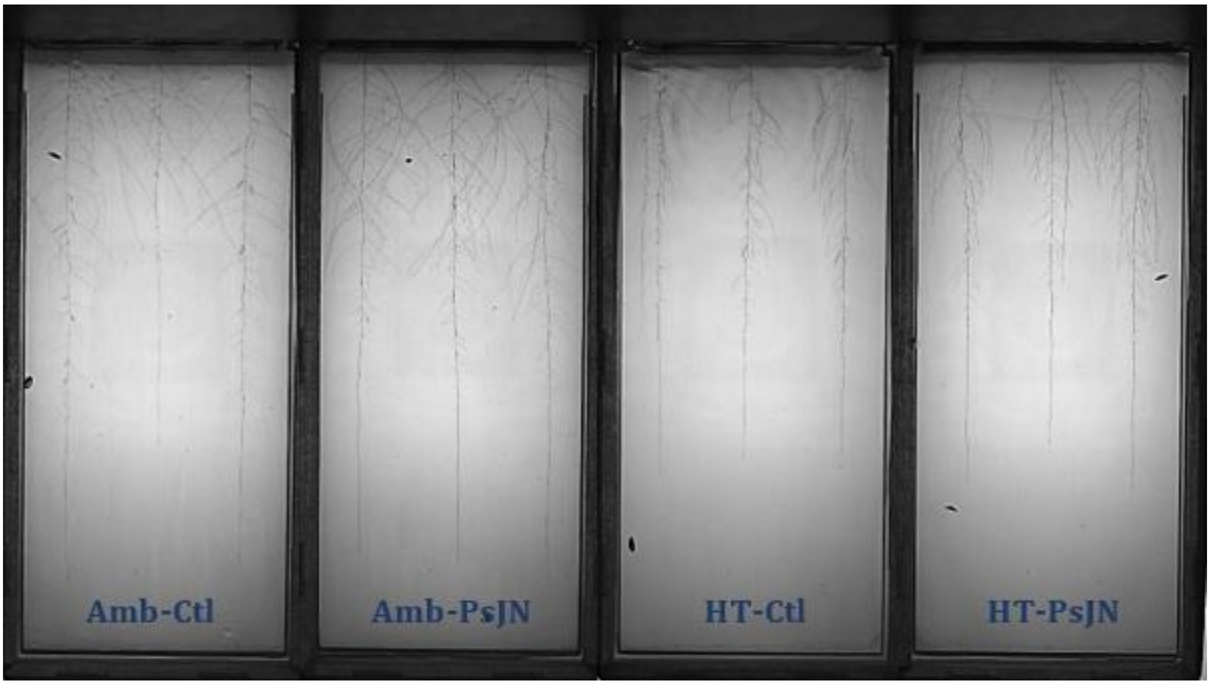

Figure S3. Sample root images generated by the GrowScreen-Agar II

Images of plant roots from the four treatments at 16 DAI taken by the root camera of the GrowScreen-Agar II imaging system. Treatments (from left to right): Amb-Ctl (control or non-inoculated plants under ambient), Amb-PsJN (PsJN-inoculated plants under ambient), HT-Ctl (control or non-inoculated plants under high temperature), and HT-PsJN (PsJN-inoculated plants under high temperature).

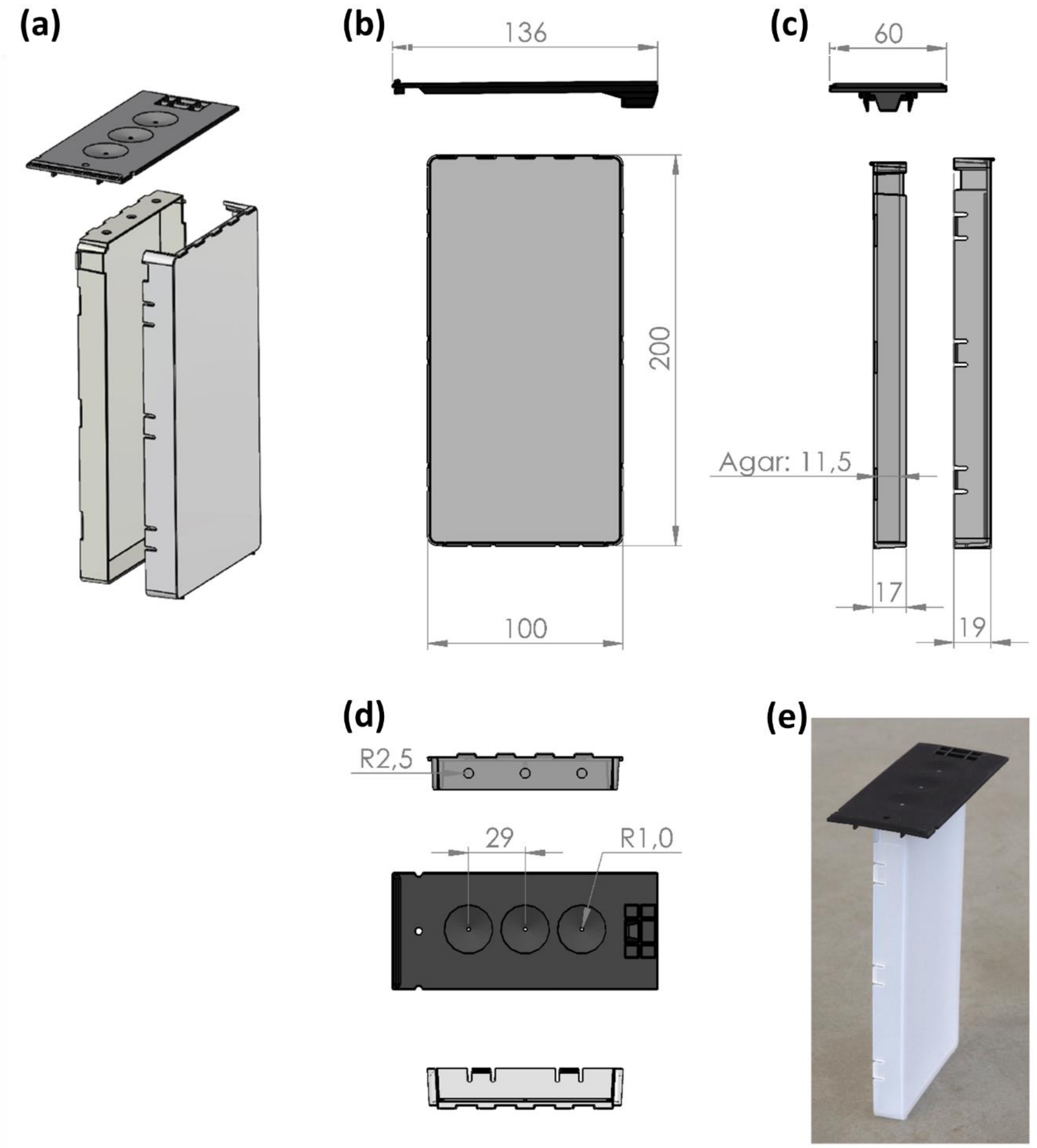

Figure S4. Agar plates for GrowScreen-Agar II

Technical drawings of three components of the agar plate: 1) an opaque cover with an anti-fog agent which prevents water droplets, 2) a transparent back plate with holes on top which allows root imaging and the shoot to grow outside of the plate, and 3) a black top part ("collar") also with three holes as background for shoot imaging, keeping light out of the root zone and mechanical support for leaves. The technical drawings show the assembly of the three components **(a)**, different side views **(b, c)**, top view **(d)**, while figure **e**) shows an original photo of the assembled plate. The dimensions are given in mm **(b-d)**. 'Agar: 11,5' represents the filling height of the agar **(c)** and 'R' the radius of the holes **(d)**.

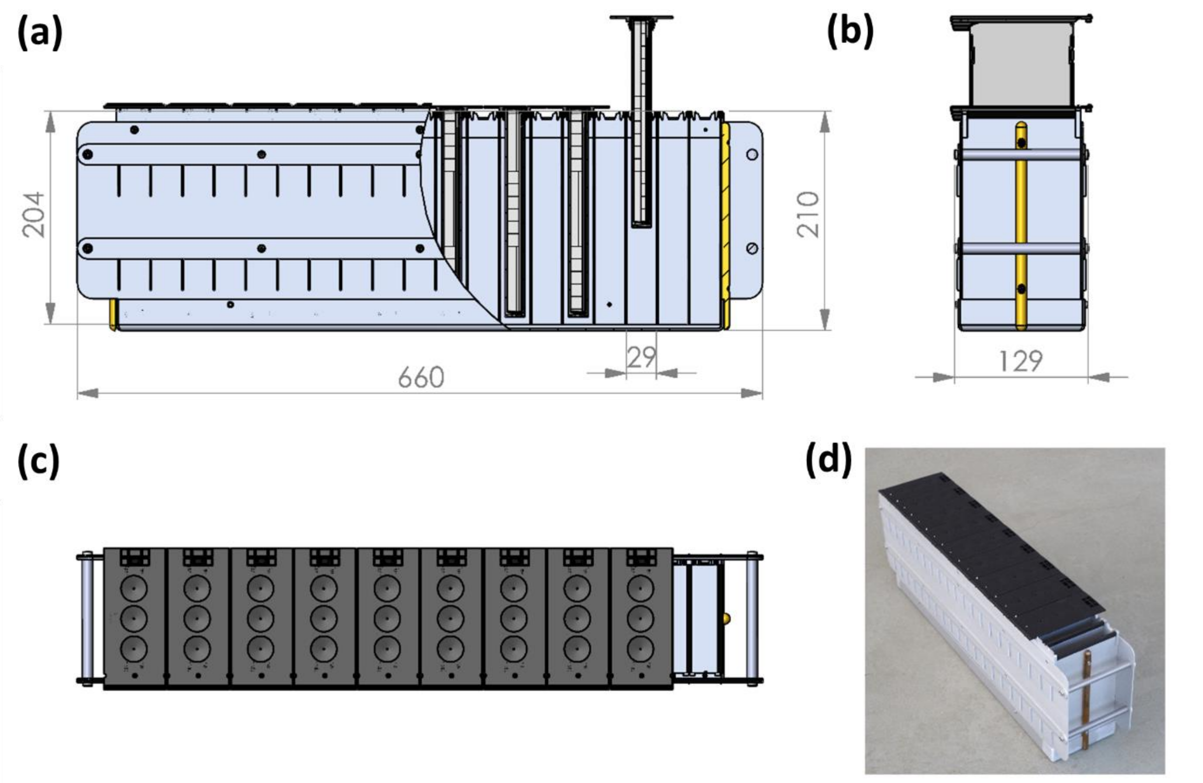

Figure S5. Magazines for GrowScreen-Agar II

The plates are positioned vertically and maintained for plant cultivation in fabricated metal magazines accommodating up to 10 plates. The design of the magazines allows the roots to grow in the dark. The technical drawings show a side view **(a)**, top view **(b)**, and front view **(c)** of the magazine, while figure **(d)** shows an original photo of the magazine, each loaded with 9 plates and 1 open slot. In figure **(a)** the side wall of the magazine is partly removed to show the plates inside the magazine and one plate is lifted in **(a)** and **(b)**. The dimensions are given in mm.

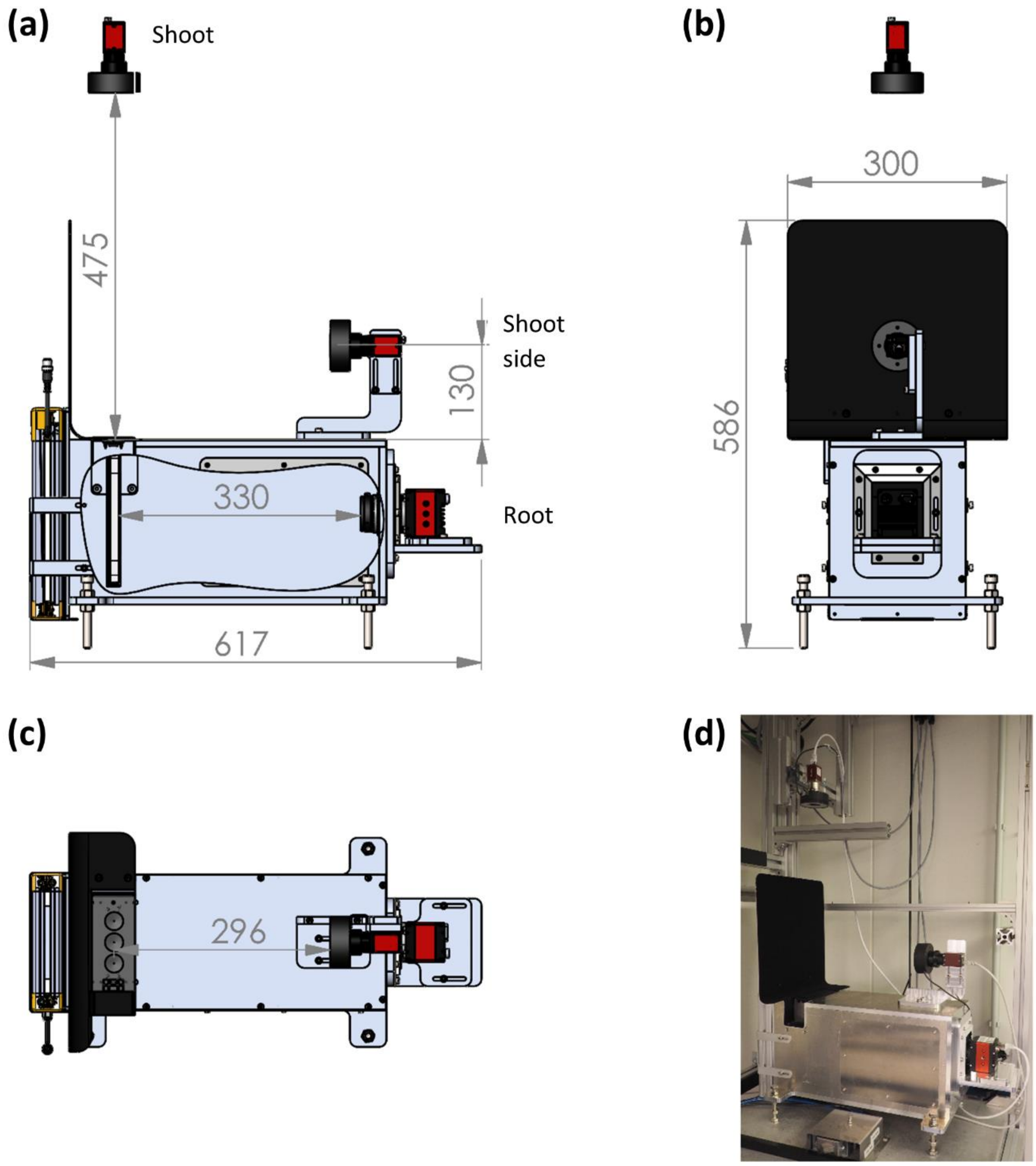

Figure S6. Imaging station of GrowScreen-Agar II

For imaging roots and shoots, the plates are placed in the imaging station of the phenotyping platform GrowScreen-Agar II. The imaging system features a metal housing equipped with one root and two shoot cameras aiming at a slot for inserting the plate. The root camera takes images of the whole agar area (20 x 10 cm) while the shoot cameras take a side view and a top view image of the shoots. The technical drawings show a side view **(a)**, top view **(b)**, and front view **(c)** of the imaging station, while the original photo shows nearly a side view **(d)**. The black background **(c, d)** provides a good contrast for shoot image analysis. Root illumination is achieved from the back while leaves are illuminated for imaging by using LED rings placed around the objective lenses of the shoot camera **(a-d)**.

Table S1. Mean values and standard error of different root type morphological traits

Root trait	DAI	Mean values ± standard error											
		Amb-Ctl			Amb-PsJN			HT-Ctl			HT-PsJN		
1. Length - Total	5	2.23	±	0.20	4.29	±	0.34	1.43	±	0.07	3.31	±	0.46
	7	3.50	±	0.29	7.08	±	0.45	1.96	±	0.11	4.39	±	0.44
	9	8.65	±	0.84	18.47	±	1.31	3.93	±	0.36	11.35	±	0.87
	12	32.41	±	3.14	65.92	±	4.37	14.06	±	1.65	42.46	±	4.13
	14	87.21	±	11.73	130.38	±	6.84	31.05	±	3.74	80.96	±	5.10
	16	212.74	±	20.75	249.04	±	11.06	61.35	±	7.62	139.61	±	9.89
	19	458.45	±	31.98	486.62	±	22.99	150.48	±	17.88	264.15	±	25.57
	21	647.27	±	40.54	798.84	±	38.75	242.07	±	27.04	366.98	±	36.10
2. Length - Primary	5	1.11	±	0.10	2.15	±	0.17	0.60	±	0.09	1.66	±	0.23
	7	1.65	±	0.12	3.53	±	0.23	0.90	±	0.10	2.20	±	0.22
	9	2.90	±	0.17	6.41	±	0.55	1.82	±	0.15	4.68	±	0.32
	12	5.26	±	0.25	10.79	±	0.64	3.64	±	0.24	10.17	±	1.11
	14	8.82	±	1.02	13.85	±	0.66	5.01	±	0.27	14.17	±	1.41
	16	12.26	±	1.20	16.70	±	0.75	6.38	±	0.27	16.44	±	1.49
	19	13.94	±	1.11	18.36	±	0.70	8.33	±	0.25	18.71	±	1.57
	21	14.02	±	1.09	18.36	±	0.70	9.45	±	0.24	19.58	±	1.56
3. Length - 1st Lateral	5	0.06	±	0.00									
	7	0.30	±	0.06	0.29	±	0.00						
	9	3.11	±	0.50	5.65	±	0.68	0.43	±	0.07	1.98	±	0.58
	12	21.78	±	2.68	44.25	±	3.73	7.40	±	1.12	22.13	±	3.59
	14	66.01	±	10.14	99.05	±	5.95	20.85	±	3.22	51.06	±	3.83
	16	165.73	±	16.26	190.65	±	9.02	47.12	±	6.87	97.05	±	7.33
	19	316.12	±	20.13	327.71	±	14.01	119.29	±	14.80	181.66	±	15.16
	21	416.80	±	24.49	451.21	±	16.32	180.06	±	19.35	236.24	±	17.69
4. Length - 2nd Lateral	5												
	7												
	9												
	12	0.23	±	0.06	0.43	±	0.07						
	14	4.27	±	1.07	3.64	±	0.43	0.45	±	0.09	1.78	±	0.89
	16	22.49	±	4.57	24.99	±	2.01	1.97	±	0.74	9.67	±	4.24
	19	114.46	±	13.24	122.19	±	9.76	14.53	±	4.64	45.07	±	13.51
	21	202.42	±	17.82	310.90	±	24.86	43.11	±	10.63	91.57	±	23.91
5. Growth rate - Total	5	0.45	±	0.04	0.86	±	0.07	0.29	±	0.01	0.66	±	0.09
	7	0.63	±	0.05	1.39	±	0.16	0.33	±	0.04	0.54	±	0.36
	9	2.57	±	0.29	5.69	±	0.56	1.06	±	0.09	3.48	±	0.40
	12	7.92	±	0.83	15.82	±	1.26	3.38	±	0.44	10.37	±	1.24
	14	27.40	±	4.75	32.23	±	1.71	8.49	±	1.09	19.25	±	0.86
	16	62.76	±	4.97	59.33	±	2.50	15.15	±	1.96	29.32	±	3.03
	19	81.90	±	4.67	118.79	±	7.39	29.71	±	3.45	41.51	±	5.71
	21	94.41	±	6.34	156.11	±	9.41	45.79	±	4.62	51.41	±	6.29
6. Growth rate - Primary	5	0.45	±	0.04	0.86	±	0.07	0.29	±	0.01	0.66	±	0.09
	7	0.54	±	0.02	1.38	±	0.16	0.33	±	0.04	0.54	±	0.36
	9	1.25	±	0.04	2.88	±	0.43	0.92	±	0.07	2.49	±	0.28
	12	1.58	±	0.12	2.92	±	0.26	1.21	±	0.07	3.65	±	0.64
	14	3.56	±	0.98	3.05	±	0.28	1.37	±	0.03	4.00	±	0.74
	16	3.44	±	0.45	2.86	±	0.28	1.37	±	0.02	2.27	±	0.27
	19	1.12	±	0.20	1.66	±	0.12	1.30	±	0.03	1.52	±	0.17
	21	1.01	±	0.00	0.04	±	0.00	1.11	±	0.03	0.87	±	0.08
7. Growth rate - 1st Lateral	5	0.01	±	0.00									
	7	0.14	±	0.03	0.15	±	0.00						
	9	1.45	±	0.23	2.81	±	0.34	0.22	±	0.03	0.99	±	0.29
	12	6.31	±	0.75	12.87	±	1.11	2.36	±	0.35	6.72	±	1.03
	14	22.11	±	4.02	27.40	±	1.51	7.04	±	1.04	14.47	±	0.71
	16	49.86	±	3.33	45.80	±	1.94	13.14	±	1.84	23.00	±	2.45
	19	50.13	±	2.31	68.53	±	3.97	24.05	±	2.69	28.20	±	3.12
	21	50.34	±	3.38	61.75	±	1.85	30.39	±	2.43	27.29	±	1.40
8. Growth rate - 2nd Lateral	5												
	7												
	9												
	12	0.08	±	0.02	0.14	±	0.02						
	14	2.08	±	0.53	1.78	±	0.21	0.22	±	0.04	0.89	±	0.44
	16	9.47	±	1.85	10.67	±	0.84	0.86	±	0.33	4.06	±	1.76
	19	30.66	±	3.04	48.60	±	4.28	4.35	±	1.34	11.80	±	3.38
	21	43.98	±	3.73	94.36	±	8.68	14.29	±	3.06	23.25	±	5.68
9. Number of 1st lateral roots	5	1.00	±	0.00									
	7	2.25	±	0.34	1.00	±	0.00						
	9	11.27	±	1.13	13.60	±	1.19	2.75	±	0.34	6.88	±	1.83
	12	32.25	±	2.94	50.20	±	3.78	17.82	±	1.83	44.25	±	6.27
	14	63.58	±	6.35	99.87	±	6.55	45.00	±	5.82	77.00	±	7.08
	16	121.42	±	9.81	159.67	±	7.95	65.08	±	7.62	119.38	±	8.57
	19	199.08	±	11.67	234.67	±	9.77	118.83	±	12.45	181.25	±	13.64
	21	238.83	±	13.23	297.07	±	10.18	152.25	±	13.89	214.88	±	14.29
10. Number of 2nd lateral roots	5												
	7												
	9												
	12	1.20	±	0.13	2.00	±	0.45						
	14	13.80	±	2.62	14.87	±	1.48	4.00	±	0.68	4.43	±	1.06
	16	46.33	±	8.09	65.07	±	4.66	10.78	±	2.81	19.63	±	5.83
	19	187.58	±	21.49	238.93	±	17.60	61.83	±	13.96	85.38	±	16.51
	21	324.67	±	29.34	605.27	±	44.47	160.75	±	29.07	181.38	±	29.63

Table S2. Mean values and standard error of different root system traits describing distribution and spread

Root trait	DAI	Mean values ± standard error											
		Amb-Ctl			Amb-PsJN			HT-Ctl			HT-PsJN		
1. Root system depth	5	2.25	±	0.20	2.32	±	0.06	1.43	±	0.07	1.59	±	0.08
	7	3.32	±	0.25	3.35	±	0.08	1.97	±	0.10	2.28	±	0.10
	9	5.83	±	0.34	5.66	±	0.12	3.55	±	0.29	4.30	±	0.14
	12	10.28	±	0.42	9.83	±	0.18	7.00	±	0.46	8.16	±	0.16
	14	13.52	±	0.46	13.08	±	0.20	9.66	±	0.52	10.96	±	0.16
	16	16.59	±	0.43	16.27	±	0.19	12.33	±	0.54	13.65	±	0.18
	19	19.02	±	0.18	19.27	±	0.02	16.10	±	0.50	17.28	±	0.24
	21	19.19	±	0.07	19.27	±	0.02	18.28	±	0.47	19.00	±	0.12
2. Root system width	5	0.11	±	0.02	0.12	±	0.01	0.08	±	0.01	0.08	±	0.01
	7	0.20	±	0.04	0.14	±	0.01	0.09	±	0.01	0.10	±	0.01
	9	0.96	±	0.13	1.25	±	0.12	0.22	±	0.04	0.37	±	0.04
	12	2.97	±	0.28	3.05	±	0.15	1.15	±	0.16	1.27	±	0.09
	14	4.21	±	0.31	4.08	±	0.18	1.87	±	0.24	2.08	±	0.15
	16	5.70	±	0.30	5.38	±	0.26	2.49	±	0.28	2.62	±	0.13
	19	7.31	±	0.26	7.00	±	0.37	3.18	±	0.28	3.45	±	0.20
	21	7.96	±	0.27	7.61	±	0.35	3.62	±	0.28	3.64	±	0.20
3. Convex hull area	5	28.47	±	3.80	28.56	±	2.20	14.00	±	1.30	18.01	±	1.96
	7	102.31	±	20.31	60.68	±	5.29	22.47	±	2.08	30.46	±	3.68
	9	904.58	±	137.17	1081.86	±	116.43	123.61	±	20.17	245.27	±	31.25
	12	4911.60	±	561.84	4832.34	±	278.58	1326.81	±	197.74	1644.98	±	140.32
	14	9749.58	±	837.04	8928.15	±	470.15	3050.44	±	409.82	3741.92	±	298.15
	16	16172.80	±	1097	15340.91	±	752.41	5362.50	±	613.27	6117.01	±	376.52
	19	26699.98	±	1041	26073.98	±	1177	9411.44	±	905.49	11132.84	±	914.97
	21	32662.63	±	954.99	32044.74	±	1336	12529.62	±	1099	13249.36	±	741.05
4. Branching angle of lateral roots	5												
	7	74.63	±	4.01	26.69	±	0.00						
	9	62.88	±	3.08	55.01	±	4.92	37.54	±	3.30	60.39	±	9.08
	12	59.10	±	2.11	59.95	±	2.03	56.13	±	3.74	55.75	±	3.84
	14	61.09	±	1.16	57.10	±	1.62	52.38	±	3.97	56.09	±	3.37
	16	61.84	±	2.94	55.05	±	1.23	55.09	±	2.70	56.69	±	2.28
	19	61.78	±	3.11	55.70	±	1.40	52.68	±	1.81	52.66	±	0.86
	21	60.54	±	2.81	54.95	±	1.38	51.73	±	1.55	50.57	±	1.09
